# The continuity of effect of schizophrenia polygenic risk score and patterns of cannabis use on transdiagnostic symptom dimensions at first-episode psychosis: findings from the EU-GEI study

**DOI:** 10.1038/s41398-021-01526-0

**Published:** 2021-08-10

**Authors:** Diego Quattrone, Ulrich Reininghaus, Alex L. Richards, Giada Tripoli, Laura Ferraro, Andrea Quattrone, Paolo Marino, Victoria Rodriguez, Edoardo Spinazzola, Charlotte Gayer-Anderson, Hannah E. Jongsma, Peter B. Jones, Caterina La Cascia, Daniele La Barbera, Ilaria Tarricone, Elena Bonora, Sarah Tosato, Antonio Lasalvia, Andrei Szöke, Celso Arango, Miquel Bernardo, Julio Bobes, Cristina Marta Del Ben, Paulo Rossi Menezes, Pierre-Michel Llorca, Jose Luis Santos, Julio Sanjuán, Manuel Arrojo, Andrea Tortelli, Eva Velthorst, Steven Berendsen, Lieuwe de Haan, Bart P. F. Rutten, Michael T. Lynskey, Tom P. Freeman, James B. Kirkbride, Pak C. Sham, Michael C. O’Donovan, Alastair G. Cardno, Evangelos Vassos, Jim van Os, Craig Morgan, Robin M. Murray, Cathryn M. Lewis, Marta Di Forti, EU-GEI collaborators, Kathryn Hubbard, Stephanie Beards, Simona A. Stilo, Mara Parellada, David Fraguas, Marta Rapado Castro, Álvaro Andreu-Bernabeu, Gonzalo López, Mario Matteis, Emiliano González, Manuel Durán-Cutilla, Covadonga M. Díaz-Caneja, Pedro Cuadrado, José Juan Rodríguez Solano, Angel Carracedo, Javier Costas, Emilio Sánchez, Silvia Amoretti, Esther Lorente-Rovira, Paz Garcia-Portilla, Estela Jiménez-López, Nathalie Franke, Daniella van Dam, Fabian Termorshuizen, Nathalie Franke, Elsje van der Ven, Elles Messchaart, Marion Leboyer, Franck Schürhoff, Stéphane Jamain, Grégoire Baudin, Aziz Ferchiou, Baptiste Pignon, Jean-Romain Richard, Thomas Charpeaud, Anne-Marie Tronche, Flora Frijda, Giovanna Marrazzo, Lucia Sideli, Crocettarachele Sartorio, Fabio Seminerio, Camila Marcelino Loureiro, Rosana Shuhama, Mirella Ruggeri, Chiara Bonetto, Doriana Cristofalo, Domenico Berardi, Marco Seri, Giuseppe D’Andrea

**Affiliations:** 1 Social, Genetic and Developmental Psychiatry Centre, Institute of Psychiatry, Psychology and Neuroscience, King’s College London, SE5 8AF, London, UK; 2 National Institute for Health Research (NIHR) Maudsley Biomedical Research Centre, South London and Maudsley NHS Foundation Trust, London, UK; 3 Central Institute of Mental Health, Medical Faculty Mannheim, University of Heidelberg, Mannheim, 68159 Germany; 4 Department of Health Service and Population Research, Institute of Psychiatry, Psychology and Neuroscience, King’s College London, De Crespigny Park, Denmark Hill, London, SE5 8AF UK; 5 Department of Psychiatry and Neuropsychology, School for Mental Health and Neuroscience, South Limburg Mental Health Research and Teaching Network, Maastricht University Medical Centre, P.O. Box 616, 6200 MD Maastricht, The Netherlands; 6 Division of Psychological Medicine and Clinical Neurosciences, MRC Centre for Neuropsychiatric Genetics and Genomics, Cardiff University, Cardiff, CF24 4HQ UK; 7 Department of Experimental Biomedicine and Clinical Neuroscience, University of Palermo, Via G. La Loggia 1, 90129 Palermo, Italy; 8 National Health Care System, Villa Betania Psychological Institute, 89100 Reggio Calabria, Italy; 9 Department of Psychosis Studies, Institute of Psychiatry, Psychology and Neuroscience, King’s College London, De Crespigny Park, Denmark Hill, London, SE5 8AF UK; 10 Psylife Group, Division of Psychiatry, University College London, 6th Floor, Maple House, 149 Tottenham Court Road, London, W1T 7NF UK; 11 Centre for Transcultural Psychiatry “Veldzicht” Balkbrug, the Netherlands, VR Mental Health Group, University Center for Psychiatry, Univerisity Medical Centre Groningen, Groningen, The Netherlands; 12 Department of Psychiatry, University of Cambridge, Herchel Smith Building for Brain & Mind Sciences, Forvie Site, Robinson Way, Cambridge, CB2 0SZ UK; 13 CAMEO Early Intervention Service, Cambridgeshire & Peterborough NHS Foundation Trust, Cambridge, CB21 5EF UK; 14 Department of Medical and Surgical Science, Psychiatry Unit, Alma Mater Studiorum Università di Bologna, Viale Pepoli 5, 40126 Bologna, Italy; 15 Section of Psychiatry, Department of Neuroscience, Biomedicine and Movement, University of Verona, Piazzale L.A. Scuro 10, 37134 Verona, Italy; 16 INSERM, U955, Equipe 15, 51 Avenue de Maréchal de Lattre de Tassigny, 94010 Créteil, France; 17 Child and Adolescent Psychiatry Department, Institute of Psychiatry and Mental Health, Hospital General Universitario Gregorio Marañón, School of Medicine, Universidad Complutense, IiSGM, CIBERSAM, C/Doctor Esquerdo 46, 28007 Madrid, Spain; 18 Barcelona Clinic Schizophrenia Unit, Neuroscience Institute, Hospital Clinic of Barcelona, Department of Medicine, University of Barcelona, IDIBAPS, CIBERSAM, Barcelona, Spain; 19 Faculty of Medicine and Health Sciences - Psychiatry, Universidad de Oviedo, ISPA, INEUROPA. CIBERSAM, Oviedo, Spain; 20 Neuroscience and Behavior Department, Ribeirão Preto Medical School, University of São Paulo, São Paulo, Brazil; 21 Department of Preventative Medicine, Faculdade de Medicina FMUSP, University of São Paulo, São Paulo, Brazil; 22 University Clermont Auvergne, CMP-B CHU, CNRS, Clermont Auvergne INP, Institut Pascal, F-63000 Clermont-Ferrand, France; 23 Department of Psychiatry, Servicio de Psiquiatría Hospital “Virgen de la Luz,”, Cuenca, Spain; 24 Department of Psychiatry, School of Medicine, Universidad de Valencia, Centro de Investigación Biomédica en Red de Salud Mental, Valencia, Spain; 25 Department of Psychiatry, Psychiatric Genetic Group, Instituto de Investigación Sanitaria de Santiago de Compostela, Complejo Hospitalario Universitario de Santiago de Compostela, Santiago, Spain; 26 Etablissement Public de Santé Maison Blanche, Paris, France; 27 Department of Psychiatry, Early Psychosis Section, Academic Medical Centre, University of Amsterdam, Amsterdam, the Netherlands; 28 Department of Psychiatry, Icahn School of Medicine at Mount Sinai, New York, NY USA; 29 National Addiction Centre, Institute of Psychiatry, Psychology and Neuroscience, King’s College London, 4 Windsor Walk, London, SE5 8BB UK; 30 Department of Psychology, University of Bath, 10 West, Bath, BA2 7AY UK; 31 Department of Psychiatry, the University of Hong Kong, Pok Fu Lam, Hong Kong; 32 Centre for Genomic Sciences, Li KaShing Faculty of Medicine, The University of Hong Kong, Pok Fu Lam, Hong Kong; 33 Division of Psychological and Social Medicine, Leeds Institute of Health Sciences, Faculty of Medicine and Health, University of Leeds, Leeds, LS2 9NL UK; 34 National Institute for Health Research (NIHR) Maudsley Biomedical Research Centre, South London and Maudsley NHS Foundation Trust, King’s College London, London, UK; 35 Brain Centre Rudolf Magnus, Utrecht University Medical Centre, Utrecht, The Netherlands; 36 Department of Health Service and Population Research, Institute of Psychiatry, King’s College London, De Crespigny Park, Denmark Hill, London, SE5 8AF UK; 37 Department of Psychosis Studies, Institute of Psychiatry, King’s College London, De Crespigny Park, Denmark Hill, London, SE5 8AF UK; 38 Department of Child and Adolescent Psychiatry, Hospital General Universitario Gregorio Marañón, School of Medicine, Universidad Complutense, IiSGM (CIBERSAM), C/Doctor Esquerdo 46, 28007 Madrid, Spain; 39 Villa de Vallecas Mental Health Department, Villa de Vallecas Mental Health Centre, Hospital Universitario Infanta Leonor / Hospital Virgen de la Torre, C/San Claudio 154, 28038 Madrid, Spain; 40 Puente de Vallecas Mental Health Department, Hospital Universitario Infanta Leonor / Hospital Virgen de la Torre, Centro de Salud Mental Puente de Vallecas, C/Peña Gorbea 4, 28018 Madrid, Spain; 41 Fundación Pública Galega de Medicina Xenómica, Hospital Clínico Universitario, Choupana s/n, 15782 Santiago de Compostela, Spain; 42 Department of Psychiatry, Hospital General Universitario Gregorio Marañón, School of Medicine, Universidad Complutense, IiSGM (CIBERSAM), C/Doctor Esquerdo 46, 28007 Madrid, Spain; 43 Department of Psychiatry, Hospital Clinic, IDIBAPS, Centro de Investigación Biomédica en Red de Salud Mental (CIBERSAM), Universidad de Barcelona, C/Villarroel 170, escalera 9, planta 6, 08036 Barcelona, Spain; 44 Department of Psychiatry, School of Medicine, Universidad de Valencia, Centro de Investigación Biomédica en Red de Salud Mental (CIBERSAM), C/Avda. Blasco Ibáñez 15, 46010 Valencia, Spain; 45 Department of Medicine, Psychiatry Area, School of Medicine, Universidad de Oviedo, Centro de Investigación Biomédica en Red de Salud Mental (CIBERSAM), C/Julián Clavería s/n, 33006 Oviedo, Spain; 46 Department of Psychiatry, Servicio de Psiquiatría Hospital “Virgen de la Luz”, C/Hermandad de Donantes de Sangre, 16002 Cuenca, Spain; 47 Department of Psychiatry, Early Psychosis Section, Academic Medical Centre, University of Amsterdam, Meibergdreef 5, 1105 AZ Amsterdam, The Netherlands; 48 Rivierduinen Centre for Mental Health, Leiden, Sandifortdreef 19, 2333 ZZ Leiden, The Netherlands; 49 Department of Psychiatry and Neuropsychology, School for Mental Health and Neuroscience, South Limburg Mental Health Research and Teaching Network, Maastricht University Medical Centre, P.O. Box 616, 6200 MD Maastricht, The Netherlands; 50 AP-HP, Groupe Hospitalier “Mondor”, Pôle de Psychiatrie, 51 Avenue de Maréchal de Lattre de Tassigny, 94010 Créteil, France; 51 INSERM, U955, Equipe 15, 51 Avenue de Maréchal de Lattre de Tassigny, 94010 Créteil, France; 52 Faculté de Médecine, Université Paris-Est, 51 Avenue de Maréchal de Lattre de Tassigny, 94010 Créteil, France; 53 Fondation Fondamental, 40 Rue de Mesly, 94000 Créteil, France; 54 CMP B CHU, BP 69, 63003 Clermont Ferrand, Cedex 1, France; 55 Université Clermont Auvergne, EA 7280, Clermont-Ferrand, 63000 France; 56 Etablissement Public de Santé Maison Blanche, Paris, France; 57 Department of Experimental Biomedicine and Clinical Neuroscience, Section of Psychiatry, University of Palermo, Via G. La Loggia n.1, 90129 Palermo, Italy; 58 Unit of Psychiatry, “P. Giaccone” General Hospital, Via G. La Loggia n.1, 90129 Palermo, Italy; 59 Departamento de Neurociências e Ciencias do Comportamento, Faculdade de Medicina de Ribeirão Preto, Universidade de São Paulo, Av. Bandeirantes, 3900 -Monte Alegre- CEP 14049-900, Ribeirão Preto, SP Brasil; 60 Núcleo de Pesquina em Saúde Mental Populacional, Universidade de São Paulo, Avenida Doutor Arnaldo 455, CEP 01246-903, Ribeirão Preto, SP Brasil; 61 Section of Psychiatry, Department of Neuroscience, Biomedicine and Movement, University of Verona, Piazzale L.A. Scuro 10, 37134 Verona, Italy; 62 Department of Medical and Surgical Science, Psychiatry Unit, Alma Mater Studiorum Università di Bologna, Viale Pepoli 5, 40126 Bologna, Italy

**Keywords:** Schizophrenia, Clinical genetics, Predictive markers

## Abstract

Diagnostic categories do not completely reflect the heterogeneous expression of psychosis. Using data from the EU-GEI study, we evaluated the impact of schizophrenia polygenic risk score (SZ-PRS) and patterns of cannabis use on the transdiagnostic expression of psychosis. We analysed first-episode psychosis patients (FEP) and controls, generating transdiagnostic dimensions of psychotic symptoms and experiences using item response bi-factor modelling. Linear regression was used to test the associations between these dimensions and SZ-PRS, as well as the combined effect of SZ-PRS and cannabis use on the dimensions of positive psychotic symptoms and experiences. We found associations between SZ-PRS and (1) both negative (B = 0.18; 95%CI 0.03–0.33) and positive (B = 0.19; 95%CI 0.03–0.35) symptom dimensions in 617 FEP patients, regardless of their categorical diagnosis; and (2) all the psychotic experience dimensions in 979 controls. We did not observe associations between SZ-PRS and the general and affective dimensions in FEP. Daily and current cannabis use were associated with the positive dimensions in FEP (B = 0.31; 95%CI 0.11–0.52) and in controls (B = 0.26; 95%CI 0.06–0.46), over and above SZ-PRS. We provide evidence that genetic liability to schizophrenia and cannabis use map onto transdiagnostic symptom dimensions, supporting the validity and utility of the dimensional representation of psychosis. In our sample, genetic liability to schizophrenia correlated with more severe psychosis presentation, and cannabis use conferred risk to positive symptomatology beyond the genetic risk. Our findings support the hypothesis that psychotic experiences in the general population have similar genetic substrates as clinical disorders.

## Introduction

The nosology of psychotic disorders relies on operationalised criteria. These criteria are based on the type and course of symptomatology and neglect the currently known risk factors for psychosis [1]. While the utility of the operationalised approach has been instrumental in standardising clinical practice and research internationally, it has also carried nosological limitations [2]. For example, the clear-cut division of non-affective and affective psychosis has been unsatisfactory both in clinical practice [3] and genetic epidemiology; indeed, the latter has consistently shown that the diagnostic categories of schizophrenia and bipolar disorder share much of their biological roots [4]. However, due to the traditional focus on diagnostic categories, questions as to whether there is a continuity of risk factors across the transdiagnostic continuum of psychosis have been marginally investigated. Therefore, an approach based on continuous, transdiagnostic symptom dimensions across the psychosis spectrum might be more appropriate to address this question [5].

Different solutions have been proposed for the structural modelling of psychopathology, including one or more factors (i.e. unidimensional and multidimensional solutions) [6]. Recently, there has been a renewed interest in the bi-factor solution [7, 8], which suits latent constructs that cannot be fully determined as unidimensional or multidimensional, as is likely to be the case in psychosis [9]. The bi-factor model of psychopathology is composed of a general factor (based on the covariance of all items) in addition to and independently from multiple specific symptom factors (based on the covariance of item sub-groups, e.g. positive, negative, disorganization, manic, and depressive items) [8, 9]. Each item loading is split between general and specific factors in a flexible way, to maximise the amount of variance absorbed by the model [10]. However, this flexibility may result in a tendency towards data overfitting and abnormal factor loadings, when compared with correlated multidimensional solutions without a general factor [10]. Nevertheless, any factor analysis carries some degree of indeterminacy in representing a theoretical construct [11], and it is assumed that all models are wrong in principle but some are useful [10]. Opting for a bi-factor solution allows to examine multidimensionality whilst retaining an important single target construct [12], such as the general factor, which is a useful representation of the common mood-psychosis spectrum in the field of affective and non-affective psychotic disorders [6, 9, 10, 13].

Within this methodological framework, we have recently investigated the relationship between a bi-factor model of psychopathology at first-episode psychosis (FEP) [14] and cannabis consumption [15]. Psychoactive compounds in recreational cannabis may elicit positive symptoms by interacting with the endocannabinoid system [16]; moreover, converging evidence suggests that cannabis users who develop psychosis have less neurodevelopmental impairments than their non-user counterparts [17]. Supporting this, in a dimensional representation of psychosis, we see that cannabis users presented at FEP with more positive and fewer negative symptoms [15], the latter considered a proxy of early neurodevelopmental impairment in psychosis [18].

Moreover, in recent years the availability of summary statistics from large genome-wide association studies (GWAS) across psychiatric phenotypes has allowed researchers to test within independent samples how the genetic liability to a disorder predicts any other traits [19]. This genetic liability can be summarised into a polygenic risk score (PRS) [19]. However, only a few studies to date have investigated the relationship between PRS and transdiagnostic symptom dimensions in psychosis, and no studies have particularly examined the general factor [20]. Three studies on schizophrenia (SZ) patients suggested that SZ-PRS correlated with negative or disorganised symptoms [21–23], which was further reported in the Psychiatric Genomics Consortium’s (PGC) large mega-analyses [24, 25]. However, other studies have not found the same pattern of associations [26, 27], and only one study reported that SZ-PRS correlated with positive symptoms [22]. Interestingly, in the general population, an association was observed between SZ-PRS and either negative [28, 29] or positive psychotic experiences [30–32]; however, negative findings have also been reported [33].

The inconsistency across studies could be explained by differences in study design, methods, GWAS power, as well as phenotypic characteristics. For example, only two studies examined patients at the FEP stage [23, 34], thus, minimising the confounding effects of antipsychotic drugs on symptoms and capturing a common comparable time point in the course of illness. Besides, most studies have not performed a factor analysis of observed symptoms to measure and validate latent constructs.

In the present study, we sought to examine the continuity of the effect of heavy cannabis use and genetic liability to psychotic disorders across the continuum of psychosis symptoms, including general and specific dimensions from a multinational sample of FEP [14] and controls representative of the population at risk [15].

Based on a priori hypotheses, we examined: (1) whether SZ-PRS was associated with (i) a higher score at the positive and negative dimensions at FEP; and (ii) a higher score at subclinical psychosis dimensions in controls; and (2) whether previously reported association of cannabis use with the positive dimensions [15] held when taking into account SZ-PRS.

## Materials and methods

### Sample design and procedures

FEP patients and population controls were recruited as part of the EUropean network of national schizophrenia networks studying Gene-Environment Interactions (EU-GEI). FEP patients were identified between 2010 and 2015 across six countries to examine incidence rates of psychotic disorders and patterns of symptomatology [35]. For examining biological and environmental risk factors, DNA samples were collected, and an extensive face-to-face assessment was conducted on 1130 FEP and 1497 controls, broadly representative of the population living in each catchment area by age, sex and ethnic group. Patients were included in the case–control study if meeting the following criteria during the recruitment period: (a) age between 18 and 64 years; (b) presentation with a clinical diagnosis for an untreated FEP, even if longstanding [International Statistical Classification of Diseases and Related Health Problems, Tenth Revision (ICD-10) codes F20–F33]; (c) residency within the catchment area. Exclusion criteria were: (a) any previous contact with psychiatric services for psychosis; (b) psychotic symptoms related to physical or neurological conditions; and (c) transient psychotic symptoms resulting from acute intoxication (ICD-10: F1x.5).

The recruitment of controls followed a mixture of random and quota sampling methods, to achieve the best possible representativeness in age, sex and ethnicity of the population living in each catchment area. The identification process varied by site and was based on locally available sampling frames, including for example, postal addresses lists and general practitioners’ lists from randomly selected surgeries. When these resources were not fully available, Internet and newspapers advertising were used to fill quotas. Exclusion criteria for controls were: (a) diagnosis of a psychotic disorder; (b) ever having been treated for psychosis. All participants provided informed written consent. Ethical approval was provided from local research ethics committees in each catchment area: South London and Maudsley and Institute of Psychiatry Research Ethics Committee; National Research Ethics Service Committee East of England–East Cambridge; Medisch-Ethische Toetsingscommissie van het Academisch Centrum te Amsterdam; Comité Ético de Investigación Clínica Hospital Gregorio Marañón; Comité Ético de Investigación Clínica del Hospital Clinic de Barcelona; Comité Ético de Investigación Clínica del Hospital Clinic Universitari de Valencia; Comité Ética de la Investigación Clínica del Principado de Asturias; Comité Ético de Investigación Clínica de Galicia; Comité Ético de Investigación Clínica del Hospital Virgen de la Luz de Cuenca; Comité de Protéction des Personnes–CPP Île de France IX; Comitato Etico Policlinico S Orsola Malpighi; Comitato Etico Azienda Ospedaleria Universitaria di Verona; Comitato Etico Palermo 1, Azienda Ospedaliera Policlinico “Paolo Giaccone”; and Research Ethics Committee of the clinical Hospital of Ribeirão Preto Medical School, University of São Paulo, Brazil.

### Measures

Data on age, sex, and ethnicity were collected using a modified version of the Medical Research Council Sociodemographic Schedule [36].

The OPerational CRITeria (OPCRIT) system [37, 38] was used to: (1) assess pre-morbid history and mental state at FEP; and (2) establish a research-based standardised diagnosis of psychotic disorder. The OPCRIT consists of a checklist that can be filled using different sources, e.g. case records or clinical interviews. Investigators’ training and monitoring was organised centrally on an online platform, which served to: implement and follow standardised procedures; provide psychopathology training; conduct all-site inter-rater reliability pre- [39] and post-training; and monitor the inter-rater reliability annually during the study [40]. All raters were included in central interrater reliability computations (k = 0.7). An additional post-training inter-reliability analysis for individual OPCRIT items was conducted by study country, which is reported in the supplementary material.

Moreover, psychopathology assessment included the use of the Schedule for Deficit Syndrome (SDS) [41] to evaluate negative symptoms, which are not extensively covered by the OPCRIT. The Community Assessment of Psychic Experiences (CAPE) [42] was administered to population controls to report their positive, negative, and depressive, psychotic experiences.

A modified version of the Cannabis Experience Questionnaire (CEQ_EU-GEI_) [43], included in the supplementary material, was used to collect extensive information on patterns of cannabis use. For the purpose of this study, we used two dichotomic variables of the questionnaire on current use and daily use of cannabis: CEQ_EU-GEI_ 15.4 (‘Do you currently use cannabis?’ Yes/no) and CEQ_EU-GEI_ 15.9 (‘How often do/did you use cannabis?’ recoded to daily use = Yes/no).

### Dimensions of psychotic symptoms and experiences

Data from OPCRIT and CAPE were analysed using item response modelling in M*plus*, version 7.4, to estimate two separate bi-factor models of psychopathology, based on the associations among observer ratings of psychotic symptoms in patients and self-rating of psychotic experiences in controls (see Supplementary Figs. S1 and S2). This methodology is described in full in earlier EU-GEI papers on transdiagnostic dimensions [14, 15]. Briefly, OPCRIT and CAPE items were dichotomised as 0 ‘*absent’* or 1 ‘*present’*, and two different bi-factor models were estimated for patients and controls. As reported in our previous publications, to ensure enough covariance coverage for item response modelling, we used the items with a valid frequency of ‘present’ ≥10% in our sample, including individuals with ≤20 missing values in the psychopathology rating. OPCRIT and CAPE data used in the analysis contained missing values, which we assumed to be missing at random, allowing for the maximum likelihood estimator to provide unbiased estimates. Bi-factor solutions were compared with three competitive solutions (i.e. unidimensional, multidimensional, hierarchical models of psychosis) using, as model fit statistics, Log-Likelihood (LL), Akaike Information Criterion (AIC), Bayesian Information Criterion (BIC), and Sample-size Adjusted BIC (SABIC), as reported in the Supplementary Table S1. McDonald’s omega (ω) [44], omega hierarchical (ω_H_) [44], and index *H* [45], were used as reliability and strength indices.

Data from SDS were analysed in M*plus*, version 7.4, following the same above-described procedure. We did not estimate a bi-factor model for SDS due to the lack of scope of testing a general factor of negative symptoms in this study. Instead, based on the structure of the negative symptom construct [46] and previous factor analysis studies on SDS [47], we estimated a multidimensional model of negative symptoms composed of the two specific dimensions of 1) ‘avolition’ and 2) (lack of) ‘emotional expressivity’ (see Supplementary Fig. S3). We considered ‘emotional expressivity’ as the most genuine phenotypic expression of primary negative symptoms for subsequent analysis, as ‘avolition’ comprises withdrawal behaviours that partly overlap with depressive symptoms or may be secondary to paranoia in a FEP sample. SDS was not administered in one of the study sites, Verona, which was therefore not included in the analysis of negative symptoms.

### Genotype procedure

The EU-GEI case–control sample was genotyped at the MRC Centre for Neuropsychiatric Genetics and Genomics in Cardiff (UK) using a custom Illumina HumanCoreExome-24 BeadChip genotyping array covering 570,038 genetic variants. Imputation was performed in the Michigan Imputation Server, using the Haplotype Reference Consortium reference panel, with Eagle software for estimating haplotype phase, and Minimac3 for genotype imputation [48–50]. The imputed best-guess genotype was used for the present analysis.

### Population stratification and polygenic risk score calculation

We performed a two-step procedure to fully account for the multi-ethnic nature of the sample (reported in full in the supplementary material), by excluding populations in our sample of very different ancestry from external European GWAS data. Briefly, as a first step, we defined individual genetic-based ancestry by merging the EU-GEI sample with the 1000 Genome Project sample phase 3 [51] and applying k-mean clustering of ancestry Principal Components (PCs) of the overlapping single nucleotide polymorphisms (SNPs). As a second step, we identified, in the EU-GEI sample, finest ancestry clusters of individuals through iterative pruning of principal component analysis (ipPCA) of SNPs, and we tested for each cluster whether SZ-PRS discriminated cases from controls (see Supplementary Fig. S4). For downstream analyses, we, therefore, merged those population clusters where (1) SZ-PRS had discriminative value and (2) European ancestry was confirmed after merging with the 1000 Genome Project sample. In the final sample (see Supplementary Fig. S5), we removed long-range genome regions with complex linkage disequilibrium (LD) patterns and constructed main SZ-PRS (see Supplementary Fig. S6). Specifically, in PRSice [52], individuals’ risk variants were weighted by the log(odds ratio), where the odds ratio was extracted from summary statistics of the PGC2 SZ mega-analysis [53], which did not include any EU-GEI sample. Logistic regression was applied to predict case status from SZ-PRS, after covarying for 10 ancestry PCs, sex, age, and primary diagnosis. To measure the variance explained by PRS, *R*^*2*^ was used as a measure of the difference in variance between the full-model versus a model with the covariates alone, at the SNPs *p*-value threshold (*P*_*T*_) = 0.05 [selected a priori as it maximised the explained variance in case status in the PGC study [53]].

### Relationship between symptom dimensions, polygenic risk scores, and cannabis use

We tested for associations between SZ-PRS and the scores on transdiagnostic dimensions of psychotic symptoms/experiences, separately in FEP and controls, using linear regression.

Specifically, in FEP, we tested for association between SZ-PRS and general, positive, negative, disorganization, manic, and depressive symptom dimensions. In controls, we tested for association between SZ-PRS and general, positive, negative and depressive psychotic experience dimensions.

To examine the combined associations of cannabis use and SZ-PRS with the positive dimensions, we used the pattern of cannabis use previously associated with the highest level of positive symptoms in our sample [15, 54], i.e. ‘daily use’ in patients and ‘current use’ in controls. We first checked for correlation with SZ-PRS, and subsequently, we added the two cannabis terms to the models. We used the likelihood ratio (LR) test to compare the model fit before and after adding cannabis use to the model.

Given the high number of outcomes (six dimensions in patients, four in controls) and predictors (SZ-PRS and cannabis use), we controlled the false discovery rate using the Benjamini and Hochberg procedure [55], tolerating a 10% false discovery rate (*q* = 0.10). Furthermore, as a sensitivity measure, in PRSice, we tested whether the effect of SZ-PRS on symptom and psychotic experience dimensions held at other *P*_*T*_ thresholds and ran a permutation analysis to control the familywise error rate further. The latter analysis was done by repeating the PRSice procedure shuffling the phenotype 5000 times to obtain an empirical distribution of the *p*-value at the best *P*_*T*_.

## Results

### Genotyped sample, population stratification and PRS computation

Differences between genotyped and not genotyped individuals in the EU-GEI case–control sample are summarised in Table 1. Population stratification findings are presented in full in the Supplementary material. Based on the case–control discriminative value of SZ-PRS in each population cluster, we analysed 1596 individuals, including 617 FEP and 979 population controls (see Supplementary Fig. S5), for whom European ancestry was confirmed using the 1000 Genome Project sample. The ability of SZ-PRS to distinguish cases from controls in the main sample is presented in the supplementary material, showing that at *P*_*T*_ = 0.05, SZ-PRS accounted for a Nagelkerke’s *R*^2^ of 0.09 (*p* = 6.9 × 10^−25^) (see Supplementary Fig. S6).

**Table 1:** Sociodemographic and clinical differences between genotyped and not-genotyped individuals.

Case/control sample *N* = 2627	GWAS—NO	GWAS—YES	Test statistics
	*N* = 556	*N* = 2,071	
(N_FEP_ = 1130, N_controls_ = 1497)	(N_FEP_ = 282, N_controls_ = 274)	(N_FEP_ = 856, N_controls_ = 1215)	
*Case/control status*			
Case	274 (49.3)	856 (41.3)	*χ*^*2*^ (1) = 11.3; *p* = 0.001
*Age*			
Mean (SD)	32.6 (11.5)	34.3 (12.4)	*t*(2,2642) = 2.9; *p* < 0.05
*Gender*			
Male	299 (53.8)	1104 (53.3)	*χ*^*2*^ (1) = 0.03; *p* = 0.84
*Self-reported Ethnicity*			
White	374 (67.3)	1520 (73.4)	*χ*^*2*^ (5 )= 8.5; *p* = 0.13
Black	78 (14)	226 (10.9)	
Mixed	54 (9.7)	172 (8.3)	
Asian	17 (3.1)	51 (2.5)	
North African	19 (3.4)	57 (2.7)	
Other	14 (2.5)	45 (2.2)	
*Country*			
United Kingdom	123 (22.1)	459 (22.2)	*χ*^*2*^ (5) = 78.7; *p* < 0.001
Holland	54 (9.7)	352 (17)	
Spain	74 (13.3)	352 (17)	
France	71 (12.8)	181 (8.7)	
Italy	157 (28.2)	310 (15)	
Brazil	77 (13.8)	417 (20.1)	
*Research Domain Criteria Diagnosis (case only sample)*			
Bipolar disorder	13 (4.7)	47 (5.5)	*χ*^*2*^ (4) = 3.3; *p* = 0.5
Major depression with psychotic features	18 (5.9)	32 (4)	
Schizophrenia	84 (30.7)	306 (35.7)	
Schizoaffective disorder	116 (42.3)	318 (37.1)	
Unspecified psychosis	48 (17.5)	148 (17.3)	

### Psychotic symptom dimensions by PRS in patients

Findings on symptom dimensions in cases by SZ-PRS at *P*_*T*_ = 0.05 are shown in Table 2. As expected in PRS cross-trait predictions [56], the magnitude of the SNPs effect was small for all the associations detected. Specifically, SZ-PRS was associated with a higher score for both the positive (B = 0.19, 95% CI 0.03–0.35; *p* = 0.019) and negative (B = 0.18, 95% CI 0.03–0.33; *p* = 0.021) symptom dimensions. We found no association between SZ-PRS and either the general factor and depressive and manic symptom dimensions.

**Table 2:** Symptom dimension scores by SZ-PRS in cases.

	General^a^	Positive^a^	Negative^b^	Disorganization^a^	Mania^a^	Depression^a^
	B (95% CI)	B (95% CI)	B (95% CI)	B (95% CI)	B (95% CI)	B (95% CI)
SZ-PRS	0.04	0.19	0.18	−0.01	0.06	−0.06
	(−0.09 to 0.18)	(0.03 to 0.35)	(0.03 to 0.33)	(−0.16 to 0.14)	(−0.07 to 0.2)	(−0.2 to 0.07)
	*p* = 0.528	***p*** = 0.019*^†^	***p*** = 0.021*^†^	*p* = 0.928	*p* = 0.378	*p* = 0.350

*B* unstandardised regression coefficient, *CI* confidence interval.

Covariates in multiple models were sex, age, 10 ancestry PCs, and categorical diagnosis.

Associations nominally significant after permutation analysis are shown in bold.

^a^Symptom dimension score from OPCRIT factor analysis.

^b^Symptom dimension score from SDS factor analysis.

*P-values nominally significant after Benjamini–Hochberg procedure,

^†^Benjamini–Hochberg *P*-value: 0.042.

**Fig. 1:**
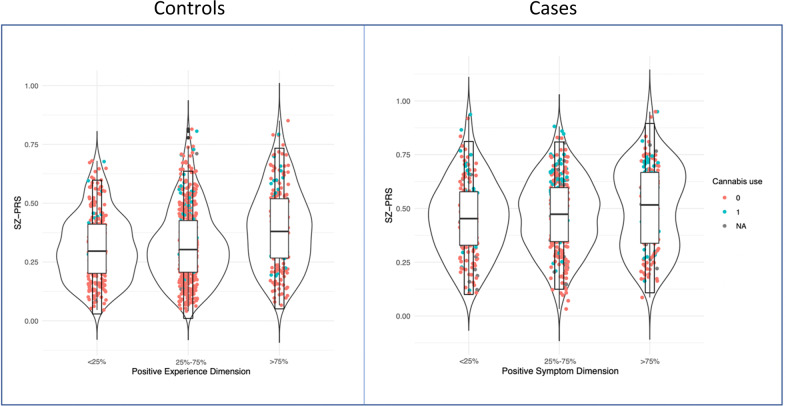
Quantiles of psychosis dimensions in the general population and separately in FEP patients by SZ-PRS. The violin plots show the distribution of SZ-PRS in the EU-GEI sample by individuals classified according to their score at the positive experience and symptom dimensions, separately in population controls (left side) and FEP patients (right side) at different quantiles (0–25% psychotic experiences or symptoms; 25–75% psychotic experiences or symptoms; 75–100% psychotic experiences or symptoms). Explanatory note: Interquartile range, 95% confidence interval, median and mean are illustrated within the bars. The shape on each side of the bars represents the density distribution. Dots indicate current cannabis use in controls and daily cannabis use in patients (red = no; green = yes).

Sensitivity analysis showed that the pattern of associations between SZ-PRS with both positive and negative symptom dimensions was consistently observed across all *P*_*T*_ and remained relevant even after permutation analysis (see Supplementary Fig. S7, showing empirical *p*-values at the best *P*_*T*_ threshold of 0.007 and 0.055 for the positive and negative symptom dimensions, respectively). The violin plots presented in Fig. 1 illustrate the distribution of predicted values of SZ-PRS after regression, across individual quantiles of positive psychotic symptoms in cases.

### Psychotic experience dimensions by SZ-PRS in controls

A positive association between SZ-PRS and a higher score at all the psychotic experience dimensions was observed (Table 3). Sensitivity analysis showed that the association between SZ-PRS with positive psychotic experiences was consistent across different *P*_*T*_ and remained relevant after permutation analysis (see Supplementary Fig. S7, showing an empirical *p*-value of 0.003). Figure 1 reports the distribution of the predicted values of SZ-PRS after regression according to individual quantiles of psychotic experiences in controls.

**Table 3:** Psychotic experience dimension scores by SZ-PRS in controls.

	General^a^	Positive^a^	Negative^a^	Depression^a^
	B (95% CI)	B (95% CI)	B (95% CI)	B (95% CI)
SZ-PRS	0.19	0.14	0.18	0.15
	(0.02 to 0.24)	(0.03 to 0.26)	(0.05 to 0.3)	(0.03 to 0.27)
	***p*** = 0.003*^††^	***p*** = 0.023*^†^	***p*** = 0.005*^††^	***p*** = 0.012*^†^

*B* Unstandardised regression coefficient, *CI* confidence interval.

Covariates in multiple models were sex, age, and ten ancestry PCs.

Associations nominally significant after permutation analysis are shown in bold.

^a^Psychotic experience dimension scores from CAPE factor analysis.

*P-values nominally significant after Benjamini–Hochberg procedure.

^†^Benjamini–Hochberg *P*-value: 0.042.

^††^Benjamini–Hochberg *P*-value: 0.027.

### Positive symptom dimensions by PRS and cannabis use in patients and controls

**Fig. 2:**
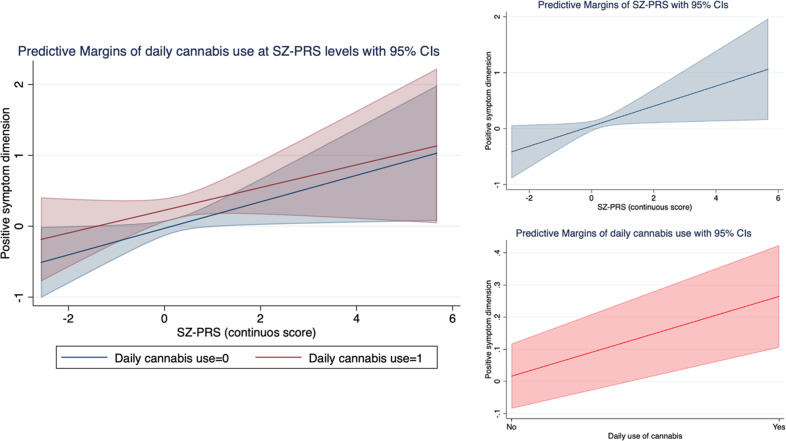
Positive symptom dimension by SZ-PRS and cannabis use in FEP patients. The graph on the left illustrates the independent and joint effect of daily cannabis use (blue line: no; red line: yes) and SZ-PRS (x axys) on the positive symptom dimension (y axys). The two graphs on the right present the main effect of SZ-PRS (in blue, x axys) and daily cannabis use (in red, x axys) on the positive symptom dimension (y axys). Values are adjusted for age, sex, and 10 ancestry PCs.

Figure 2 shows that daily cannabis use (B = 0.31; 95%CI 0.11–0.52; *p* = 0.002) and SZ-PRS (B = 0.22; 95%CI 0.04–0.39; *p* = 0.014) were independently associated with the positive symptom dimension in patients, and this joint model improved fit over a model with SZ-PRS alone (LR chi2(1) = 6.10, *p* = 0.01).

Similar results were found for the positive psychotic experience dimension in controls, with main effects of current use of cannabis (B = 0.26, 95%CI 0.06– 0.46; *p* = 0.011) and SZ-PRS (B = 0.13, 95%CI 0.02–0.25; *p* = 0.022), showing an improvement of the model fit (LR chi2(1) = 6.42, *p* = 0.01).

## Discussion

### Principal findings

This is the first study to investigate the effects of SZ-PRS and cannabis use on the psychosis dimensions in a FEP case–control sample. We found that these two factors, independently from each other, are associated with more clinical and sub-clinical positive symptoms in both FEP patients and controls. Moreover, we found a relationship between SZ-PRS and more clinical and sub-clinical negative symptoms.

Our findings provide evidence that in both patients and controls, SZ risk variants and cannabis use map onto the latent structure of psychopathology, which was built using a statistically guided approach. This supports the validity and utility of the symptom dimension approach. Further interpretation of the clinical application of these findings should take into account the small magnitude of the detected associations.

### Comparison with previous research

Our findings extend previous research on the validity of the psychosis symptom dimensions by determining their relationship with genetic factors and cannabis use. Supporting the hypothesis that symptom presentation is partly a function of SZ genetic liability, we reported an association between SZ-PRS and both positive and negative symptom dimensions. This is in line with a meta-analysis suggesting that different SZ risk loci impact on SZ clinical heterogeneity, e.g., genes related to immune system might be overrepresented for negative symptoms, and genes related to addiction and dopamine-synapses might be overrepresented for positive symptoms [57].

Familial co-aggregation of negative symptoms was reported in the Danish adoption study [58], in the Roscommon family study [59], and suggested in the Maudsley twin series studies [60]. Genome-wide suggestive linkages with an effect on negative symptoms have also been reported, although without reaching a significant threshold [61, 62]. GWAS and PRS examinations provide adequate evidence of a polygenic signal for negative symptoms [21, 22, 24, 25, 63]. Altogether, these studies indicate that the negative symptom dimension has substantive heritability, and this may be partly due to cumulative schizophrenia risk loci. The disorganization dimension has also been reported as having high heritability in some studies [60, 64], but we found no evidence of its association with SZ-PRS in our FEP sample. The prevalence of disorganization symptoms may differ in FEP and chronic patients. Furthermore, genetic loci impacting on the disorganization dimension may be different from those carrying a SZ risk [64], however, this remains speculative.

Our results on the relationship between SZ-PRS and the positive symptom dimension are less consistent with previous literature. Familial co-aggregation of positive symptoms was rarely reported [65, 66]. However, a previous study observed that, in patients with bipolar disorder, a higher SZ-PRS correlated with mood-incongruent positive symptoms [67]. Nevertheless, this was not confirmed by a meta-analysis of schizophrenia PGC and GPC samples [25, 68]. Whereas in the current study, the EU-GEI sample consisted of FEP patients; hence symptomatology rating may have been less confounded by antipsychotic treatment. On the other hand, PGC and GPC included chronic schizophrenia samples, where long-term antipsychotic treatment could attenuate positive symptoms and worsen negative symptom presentation (i.e. secondary negative symptoms). Moreover, various environmental factors may impact at different levels on endocannabinoid and dopaminergic activity, making it difficult to disentangle the risk variants contribution to positive symptoms over the course of SZ.

In the current study, we replicated the patterns of associations between SZ-PRS and psychosis dimensions as seen in cases in the control sample, in the form of sub-clinical psychosis. Our findings support previous evidence that SZ-PRS correlates with psychotic experiences that in adults may be reflecting similarities with biological SZ risk factors [31]. This is in line with the theory that psychosis is distributed as a continuum [69].

Interestingly, the general factor correlated well with the SZ genetic liability in controls but not in patients. These findings are in line with the view that psychosis exists on a continuum and general psychotic experiences though not fully shaped can be experienced by general population [70]. This is further in support of a general psychosis factor being a useful and valid phenotype in the general population [71, 72]. However, it must be acknowledged that the general factor may vary in its structure and interpretation [73], and the negative finding in cases may be explained by this factor not exactly reflecting the full range of general psychopathology in our FEP sample, as we have previously reported [14].

Furthermore, while we found the most severe level of positive symptoms at FEP among cannabis users with a high SZ-PRS, our data clarify that cannabis use is associated with more positive symptomatology [15, 54, 74] independently of genetic risk. This is especially important as the phytocannabinoids, contained in cannabis, exert their psychoactive effects acting on the endocannabinoid system, which is in turn influenced by many other biological pathways [75]. Moreover, our group has previously shown that exposure to cannabis accounts for a substantial proportion of new cases of psychosis across Europe [43]. Present findings further suggest, in a transdiagnostic fashion, that exposure to cannabis is associated with experiencing more psychotic symptoms at FEP independently from the genetic liability to SZ and regardless of being a case or a control. While only a small proportion of cannabis users develop a full-blown psychotic disorder, our results indicate that cannabis use plays an independent role from SZ genetic liability in shaping psychopathology at psychosis onset.

### Limitations

Our findings should be considered in the context of the following limitations.

1. We performed extensive work for defining the fine-scale population structure in a multi-ethnic sample. Indeed, having a sample of individuals from a single homogenous population might have improved the quality of the analysis. However, our study has the advantage of being more representative of real clinical practice. Most important, we included as far as possible population clusters not located in Europe but still suitable for PRS analyses, which is in line with the ethical aim of trying to not contribute to health disparities [76].
2. Regarding symptom ratings in patients, we used symptom dimensions from two different scales, i.e., negative from SDS, and the other symptom dimensions from OPCRIT. In the EU-GEI study, negative symptoms were accurately rated through the administration of SDS; moreover, exploratory factor analyses of OPCRIT in other samples showed that a hybrid disorganised/negative dimension was often obtained rather than discrete negative and disorganised dimensions [25, 77]. Of note, our preliminary analysis of SZ-PRS and negative dimension using OPCRIT showed no nominal association [78], due, possibly, to the scarce item covariance coverage, acknowledged as a limitation in our earlier paper on symptom dimensions [14].
3. Regarding the bi-factor solutions, the general factor may be difficult to interpret and possibly overfits the data [79]. Based on the strength of item factor loadings in our sample, the general factor could be interpreted: (1) in patients, as combined manic-delusional symptomatology [14]; (2) in controls, as a composite measure of all types of psychotic experiences [15, 54]. Moreover, in our model, the general factor may improve the measurement of specific dimensions by making their score not unduly affected by all-item covariance [14].
4. We did not validate self-reported information on the current use of cannabis with biological samples. However, this method does not allow ascertaining lifetime patterns of cannabis use [43] and is not considered a gold standard methodology [80]. Moreover, it has been shown that self-report information on cannabis use is consistent with laboratory data [81].
5. We did not use a PRS based on GWAS of symptom dimensions, as this is currently unavailable. It is noteworthy that, genes conferring risk to a disorder (‘risk genes’) may not overlap with genes modifying symptom presentation (‘modifier genes’) [82], although it is hypothesised that there are genes with a mixed effect [57]. Thus, our study answers the question whether the genetic liability for SZ due to common variants explains variance of some phenotypic traits, without accounting for other possible genetic sources of that variance (i.e. the contribution of modifier genes, copy number variants, and rare variants).
6. Finally, SZ-PRS could increase the risk for positive symptoms in cases and psychosis experiences in controls, without there being a unique continuous dimension of symptoms between the two groups. However, we could not examine cases and controls together, as two different scales were administered for psychosis rating.

### Implications

Clinicians and researchers continue to debate the validity of psychiatric nosology. We provide evidence that the bi-factor model of psychopathology is a valid instrument toward conducting high-quality transdiagnostic research into psychosis. Although PRSs are not yet applicable in clinical practice, they may serve to validate theoretical constructs. Furthermore, these findings reinforce the case for using symptom dimension ratings into routine clinical practice, which may integrate our traditional diagnostic categories. They also inform that the risk of experiencing positive psychotic symptoms associated with cannabis use is independent from individual genetic susceptibility to schizophrenia. Finally, acknowledging the impact of cannabis use, especially daily use, on symptoms presentation at first onset psychosis can guide the development of tailored intervention for those patients who continue to use cannabis following their illness onset.

## Supplementary information

Supplemental material
