# Supplementary material for: The continuity of effect of schizophrenia polygenic risk score and patterns of cannabis use on transdiagnostic symptom dimensions at first-episode psychosis: findings from the EU-GEI study
Source: Transl Psychiatry. 2021 Aug 10;11:423. doi: 10.1038/s41398-021-01526-0 (PMC8355107; doi:10.1038/s41398-021-01526-0)
Supplement: Supplementary file 1 — Supplemental material [file 41398_2021_1526_MOESM1_ESM.pdf]

Supplementary material

Item response modelling ... 2
Table S1. Model fit statistics of unidimensional, multidimensional, bifactor, second-order models for psychotic symptoms and experiences. ... 2
Figure S1. Bifactor model of psychopathology in FEP patients based on OPCRIT items. 3
Figure S2. Bifactor model of psychotic experiences in population controls based on CAPE items. ... 3
Figure S3. Multidimensional model of negative symptoms at FEP based on SDS items. ... 4
Examination of cross-national OPCRIT inter-rater reliability and measurement invariance ... 5
Cross-national OPCRIT inter-rater reliability ... 5
Cross-national measurement invariance ... 5
Population structure and PRS examination ... 6
Methods ... 6
Determination of genetic-based ancestry ... 6
Determination of the fine population structure using iterative pruning ... 6
Computation of cluster-specific principal components and PRSs ... 6
Computation of main principal components and PRSs across populations ... 6
Sensitivity analyses ... 7
Results ... 7
Sample and Genotype Quality Control (QC) ... 7
Population structure ... 7
Main PCs and PRS computation ... 8
Figure S4. Identified population subgroups and related SZ-PRS. ... 9
Figure S5. Cases and controls distribution across population clusters. ... 15
Figure S6. SZ-PRS in the final sample at different Pt-thresholds. ... 18
Figure S7. SZ-PRS by positive and negative symptom dimensions in patients and controls. ... 19
Appendix, the Cannabis Experience Questionnaire used in the EU-GEI study ... 23

Item response modelling

Table S1. Model fit statistics of unidimensional, multidimensional, bifactor, second-order models for psychotic symptoms and experiences.

OPCRIT (PATIENTS) (1)				
	Full information fit statistics[a]			
	LL	AIC	BIC	SABIC
A - Unidimensional Model	-29965	60126	60618	60306
B - Multidimensional Model (five uncorrelated factors)	-28070	56335	56826	56515
C - Multidimensional Model (five correlated factors)	-27894	56004	56546	56202
**D - Bifactor Model (one general factor and five specific uncorrelated factors)**	**-27597**	**55489**	**56226**	**55759**
E - Hierarchical Model (five first-order specific correlated factors and one second order general factor)	-27995	56197	56713	56386
CAPE (CONTROLS) (2)				
	Full information fit statistics[a]			
	LL	AIC	BIC	SABIC
A - Unidimensional Model	-23638	47397	47715	47524
B - Multidimensional Model (three uncorrelated factors)	-23844	47808	48126	47936
C - Multidimensional Model (three correlated factors)	-23341	46808	47142	46942
**D - Bifactor Model (one general factor and three specific uncorrelated factors)**	**-23139**	**46458**	**46935**	**46649**
E - Hierarchical Model (three first-order specific correlated factors and one second order general factor)	-23341	46807	47135	46938

LL, log-likelihood; AIC, Akaike Information Criterion; BIC, Bayesian Information Criterion; SABIC Sample-size Adjusted Bayesian Information Criterion
A difference of 10 in AIC, BIC and SABIC is considered important. Lower values indicate a statistically better model fit (best values across models are indicated in bold).

Figure S1. Bifactor model of psychopathology in FEP patients based on OPCRIT items.

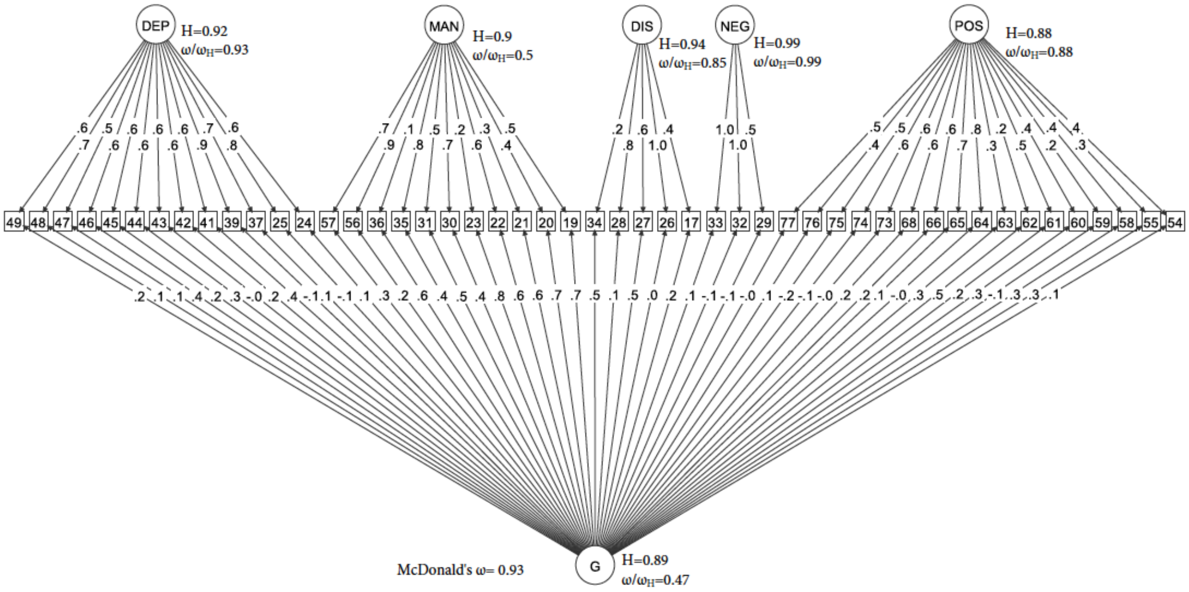

Figure S2. Bifactor model of psychotic experiences in population controls based on CAPE items.

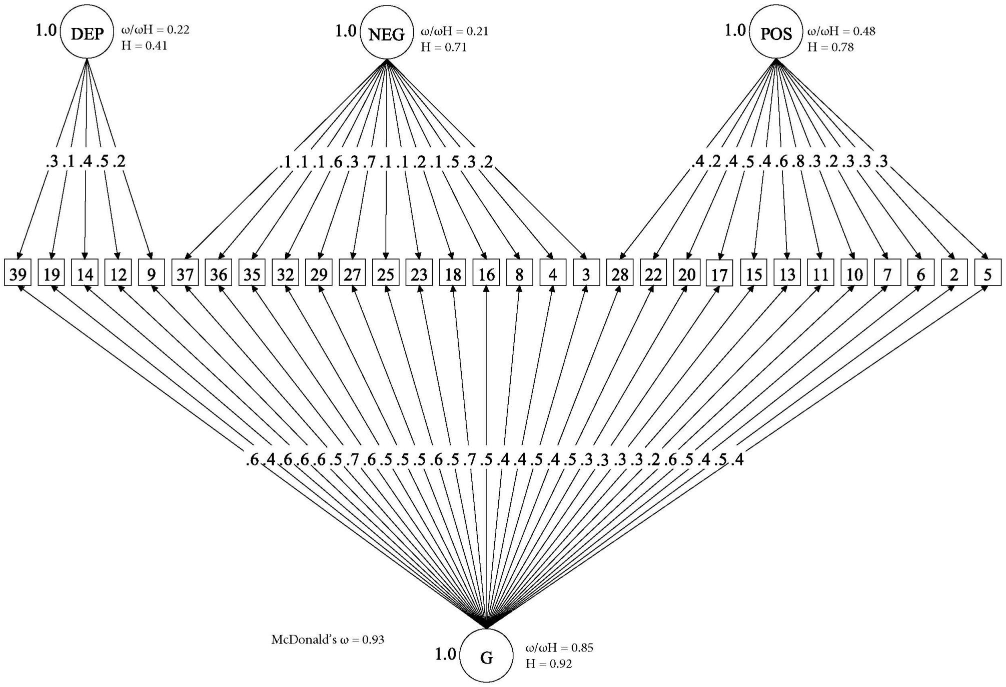

Figure S3. Multidimensional model of negative symptoms at FEP based on SDS items.

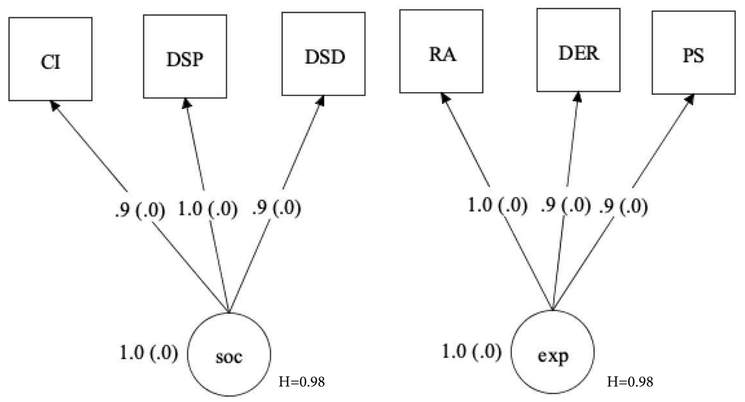

(☐) Observed variables (SDS items); (○) Unobserved variables (latent factors); (→) standardized item loading estimation onto latent factors; soc, avolition factor (e.g., active social withdrawal); exp, emotional expressivity factor. SDS items: CI, curbing of interests; DSP, diminished sense of purpose; DSD, diminished social drive; RA, restricted affect; DER, diminished emotional range; PS, poverty of speech.

H=construct reliability index; *H* is an index of the quality of the measurement model based on the loading of SDS items into each dimension (3). Indices can range from 0 to 1, with values closer to 1 indicating a better construct reliability and replicability across studies.

Examination of cross-national OPCRIT inter-rater reliability and measurement invariance

Cross-national OPCRIT inter-rater reliability

Investigators' training and monitoring was organised centrally on an online platform, which served to: implement and follow standardised procedures; provide psychopathology training; conduct all-site inter-rater reliability pre- (Berendsen et al., 2020) and post- training; and monitor the inter-rater reliability annually during the study (Gayer-Anderson et al., 2020). All raters were included in central interrater reliability computations. In addition, we have calculated post-training inter-rater reliability for individual OPCRIT items within countries, finding the following values:

United Kingdom, K-alpha 0.9085, 95% CI 0.8191 – 0.9760.
The Netherlands, K-alpha 0.7770, 95% CI 0.7311 – 0.8205.
France, K-alpha 0.7711, 95% CI 0.5739 – 0.9297.
Spain, K-alpha 0.7679, 95% CI 0.6550 – 0.8495.
Italy, K-alpha 0.5274, 95% CI 0.4926 – 0.5607.

Noteworthy, the above agreement statistics refer to the OPCRIT item level, which are usually lower than the OPCRIT diagnostic level (Brittain et al., 2013). We could not calculate inter-rater reliability for Brazil, as only one rater in Brazil completed the central training and was in charge of OPCRIT administration.

The lower interrater K-alpha for Italy may be due to the higher number of Italian raters involved in OPCRIT training and administration compared with other countries. However, this moderate value is in line with other studies. A review on OPCRIT reliability showed that, in six studies that provided the agreement between raters at the OPCRIT individual item level, the mean inter-rater kappa was 0.69 with a range from 0.48 to 0.84 (Brittain et al., 2013). Nevertheless, we have run a sensitivity analysis on our main findings, which confirmed that the independent effect of SZ-PRS (B=0.24 [0.05 to 0.43, p=0.014]) and daily cannabis use (B=0.24 [0.04 to 0.45, p=0.021]) on the positive symptom dimension held even excluding Italy (sample size, N=530).

Cross-national measurement invariance

To ensure that the OPCRIT bifactor model had an equivalent cross-country structure and its latent factors measured psychopathology with equivalence, we examined the measurement invariance in our sample. Hence, grouping by country, we estimated the multigroup invariance of the bifactor model in Mplus. Specifically, we measured 1) the configural invariance, to examine the hypothesis that the bifactor structure did not substantially differ across countries; and, subsequently, 2) the scalar invariance, to examine the hypothesis that differences in factor scores among individuals were equivalent in different countries. We specified a theta parameterization, and weighted least squares as the estimator, and we used the following goodness-of-fit criteria: comparative fit index (CIF); Tucker-Lewis index (TLI); and the root mean square error of approximation (RMSEA). Cut-off values indicating at least an adequate fit for these indices are: CFI ≥.90; TLI ≥ 0.90; and RMSEA <.08 (van de Schoot et al., 2012).

We found that the configural invariance model had an adequate fit, indicating that there was enough equivalence in the bifactor structure across countries (CFI=0.94; TLI=0.933; RMSEA 0.055 [90%CI 0.054 to 0.056]). We found changes in goodness-of-fit criteria between the configural and the scalar invariance models. However, the model fit of the scalar invariance model remained adequate (CFI=0.901; TLI=0.902; RMSEA 0.066 [90%CI 0.065 to 0.068]), which suggests that the hypothesis of scalar equivalence across countries should not be rejected.

Population structure and PRS examination

Methods

We used a step-by-step approach to account for the multi-ethnic structure of the EU-GEI sample, as follows.

Determination of genetic-based ancestry

We firstly merged our sample with the 1000 Genome project sample phase 3 (4) to build ancestry Principal Components (PCs) of the overlapping SNPs. Subsequently, we applied k-mean clustering determining individual superpopulation (EUR, AMR, SAS, EAS, AFR) in our sample based on 1000 genome sample information.

Determination of the fine population structure using iterative pruning

We used unsupervised clustering based on iterative pruning of single nucleotide polymorphisms (SNPs) in R, using the 'IPCAPS' package (5), to capture the fine-scale population structure. This method involves repetitive splits based on multivariate Gaussian mixture modelling of principal components (PCs). Unlike admixture profiling procedures, we did not make assumptions on the population ancestry in our sample, so the algorithm freely assigned individuals to population on nested datasets until no further population substructure was identified. We regulated the fixation index (F_{ST}) at 0.008, which is the common measure of the genetic distance between populations (4).

Computation of cluster-specific principal components and PRSs

Genotype pre-processing steps were repeated for each identified population cluster, based on the assumption that allele frequencies vary across populations. Specifically, using plink 1.9, in each sub-population we excluded SNPs with minor allele frequency (MAF) <0.05%, Hardy Weinberg Equilibrium $p<10^{-6}$, missingness >2%, and subjects with heterozygosity F_{het} >0.14 or <-0.11 or relatedness >0.1. Within each population cluster, we built ancestry principal components and SZ-PRS, with the aim to examine the predictive value of cluster-specific SZ-PRS and identify the suitable main sample for subsequent analysis. All retained individuals were European according to the ancestry determination based on the 1000 genome sample data.

Computation of main principal components and PRSs across populations

For constructing main PCs in the final sample, we pre-processed SNPs by using clumping which retains the *'index'* SNP for each linkage disequilibrium (LD) region (i.e., the SNP with the highest MAF), using the R packages 'bigsnpr' and 'bigstatsr' (6). Further, LD pattern differs by population, due to biological events such as, for example, inversion polymorphism (7). Thus, to ensure that main PCs did not capture mainly the variance due to differences in long-range genome regions with complex LD patterns, we ran an iterative algorithm to identify and remove these regions within our sample (6). Finally, we repeated the PRSice procedure in the main sample and built SZ-PRS, using the PGC2 GWAS as training set (8).

Sensitivity analyses

Given that, in the EU-GEI sample, controls are representative of the general population and not matched with cases, two sensitivity analyses were performed to verify that case-control PRS prediction was not affected by differences in case-control ancestry distribution. First cases and controls were plotted in the PCA multidimensional space, within and across population nodes, to exclude systematic differences in their distribution at the visual inspection. Second, controls were formally matched with cases by sex and age-range within population nodes, and SZ-PRS case-control prediction was performed in a final matched case-control sample.

Results

Sample and Genotype Quality Control (QC)

We recruited 1,130 FEP case participants and 1,497 control participants, who were assessed face-to-face. We successfully genotyped 76% of FEP (*N*=856, source of sample: 85.6% blood and 14.4% saliva) and 81% of controls (*N*=1,215, source of sample: 84.7% blood and 15.3% saliva). After imputation, 8,277,535 variants with info score > 0.6 were identified. Genotyped individuals were more likely to be older and of a white ethnicity than those not genotyped. Buccal sample collection was used more frequently in older individuals or in those of black/mixed ethnicity.

Population structure

Iterative pruning led to the identification of three first-degree nodes.

Node no.1 had three second-degree sub-nodes (Figure S4, sub-nodes A, B, and C), comprising White-British individuals recruited in the UK and Dutch individuals recruited in Holland (sub-node A); Spanish individuals recruited in Spain (sub-node B), and Italian individuals recruited in Italy (sub-node C).

Node no.2 had two main sub-nodes (Figure S4, sub-node D). Sub-node D was composed of White Brazilian ethnicity, with a smaller representation of Brown Brazilian ethnicity, recruited in Brazil; whereas sub-node D_1 was composed of individuals of Arab/Maghreb ethnicity recruited especially in France. As sub-node D_1 was relatively small, we merged it into sub-node D.

Node no.3 had two second-degree sub-nodes (sub-nodes E and F). Sub-node E was mainly composed of Asian and South American individuals recruited across different countries, and Surinamese individuals recruited in Holland. Sub-node F was composed mainly of Black African, Black Caribbean, and Brown or Black Brazilian individuals, most recruited in the UK or Brazil.

At P_T=0.05, SZ-PRS was associated with case status in 5 out of 6 population clusters (sub-nodes A-E), explaining up to 13% of the variance in sub-node D.

Sensitivity analyses excluded that the ancestry distribution of cases and controls in this sample could have a substantial impact on PRS prediction. More specifically, first, there were no observable

systematic differences in the case-control distribution in the PCA ancestry multidimensional spaces (Figure S5). Second, SZ-PRS case-control prediction in the final matched sample, composed of =521 pairs of cases and controls (N=179 from sub-node A; N= 113 from sub-node B; N=106 from sub-node C; and N=123 from sub-node D), accounted for a Nagelkerke's R2 of 0.094 ($p=8.4x10^{-17}$) at the fixed Pt-threshold of 0.05. These results were fully consistent with the main analysis in 617 cases and 979 unmatched controls (Nagelkerke's R2 = 0.09; $p=6.9x10^{-26}$), at the same fixed Pt-threshold of 0.05.

Main PCs and PRS computation

Based on the case-control discriminative value of SZ-PRS, we merged 1,612 individuals clustered in nodes from A to D (1,596 had psychosis dimension scores and full set of covariates available for the main analyses). Five long-range LD regions on chromosome 6 (from 29155749 to 30578335; from 31386313 to 31978687; from 32804798 to 33460609; from 33841361 to 34455330; from 35377301 to 36288879) and one long-range LD region on chromosome 8 (from 15773120 to 19548644), were identified in the main sample and removed for computing the main PCs. A breakdown of self-reported ethnicity by each population cluster in the final sample is reported in the Supplementary Table S2. Figure S6 shows SZ-PRS case control prediction in the final sample.

Figure S4. Identified population subgroups and related SZ-PRS.

For each population cluster (e.g., six sub-nodes labelled from A to F), the first plot shows three ancestry principal components by individual self-reported ethnicity; and the second plot shows the variance in case-control status (y-axis) explained by SZ-PRS at different P_T (x-axis). The gradient of colour of the bars represents the significance of the association; however, a fixed P_T =0.05 was *a-priori* selected for the main analysis. Ethnic group abbreviations are reported at the end of the supplementary material.

A) Sub-node A (most represented population: 40% White-Dutch (WD) recruited in Holland; 38% White-British (WB) recruited in UK; 11% White-French (WF) recruited in France)

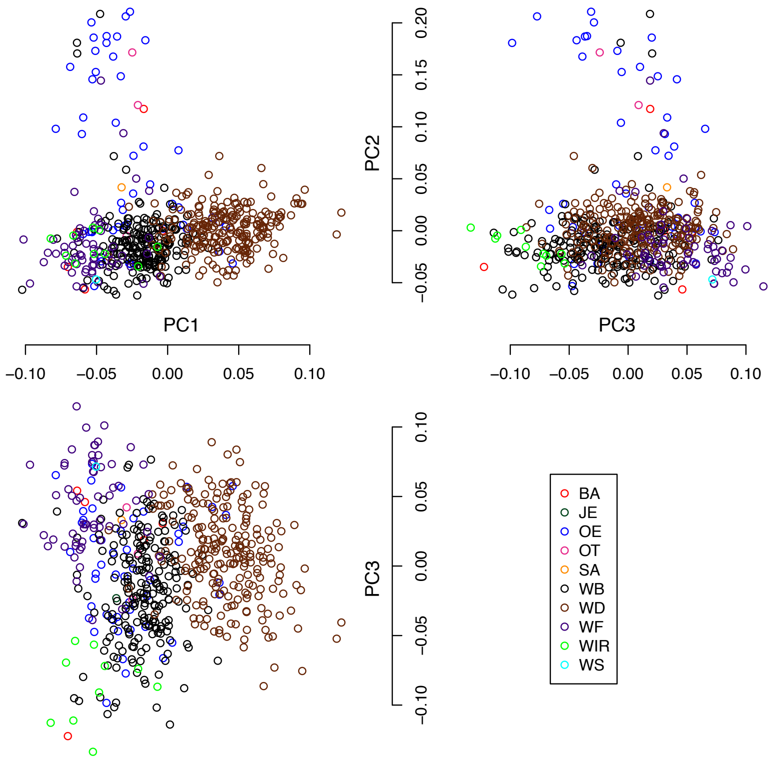

A) SZ-PRS, at Pt=0.05: Nagelkerke's R^2=0.076; p=4.1x10^{-8}

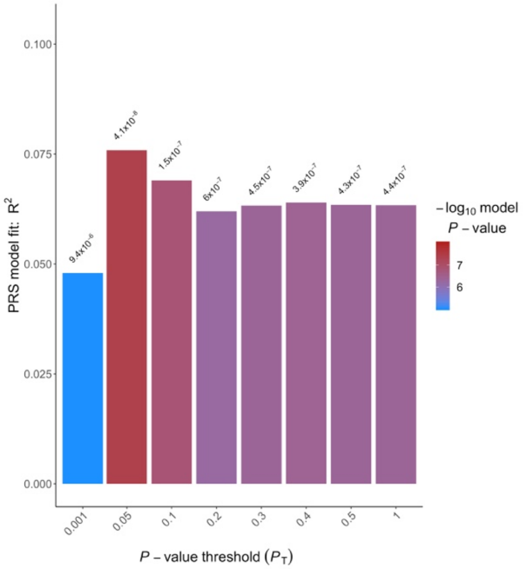

B) Sub-node B (most represented population: 84% White-Spanish (WS) recruited in Spain; 8% White-French (WF) recruited in France; 2% White-Brazilian (WB) recruited in Brazil)

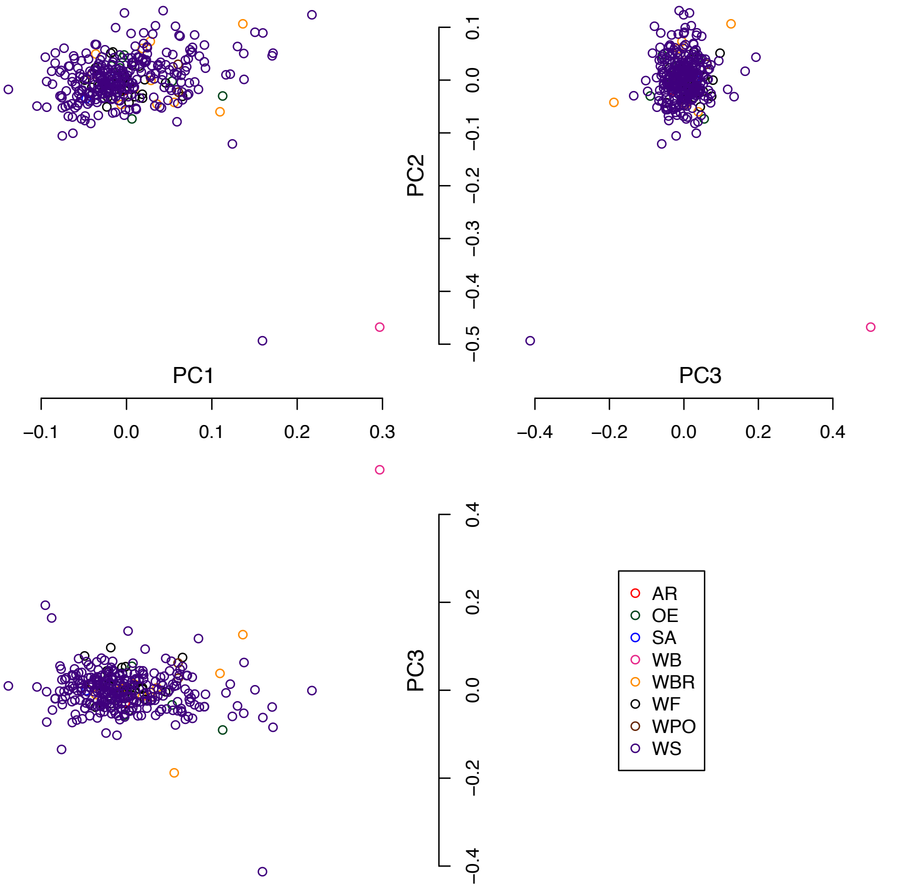

B) SZ-PRS: at Pt=0.05, Nagelkerke's R^2=0.11; p=5.2×10^{-7}

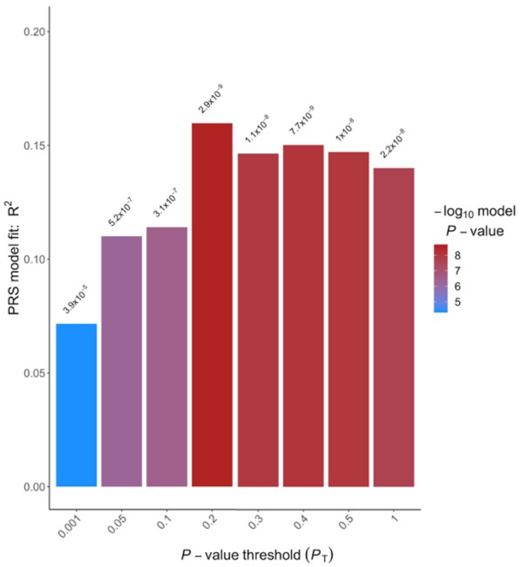

C) Sub-node C (most represented population: 77% White-Italian WI) recruited in Italy; 11% White-Brazilian (WBR) recruited in Brazil; 3% White-French (WF) recruited in France)

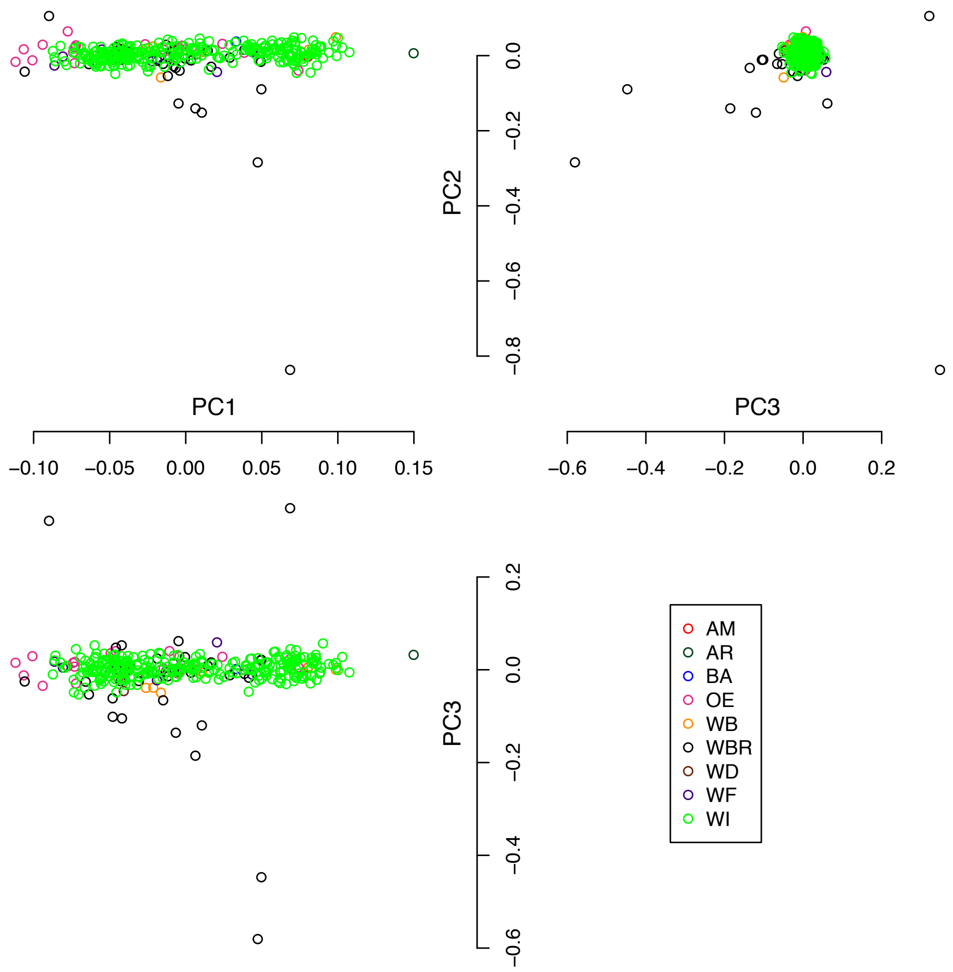

C) SZ-PRS - at Pt=0.05, Nagelkerke's R^2=0.052; p=$2.3x10^{-4}$

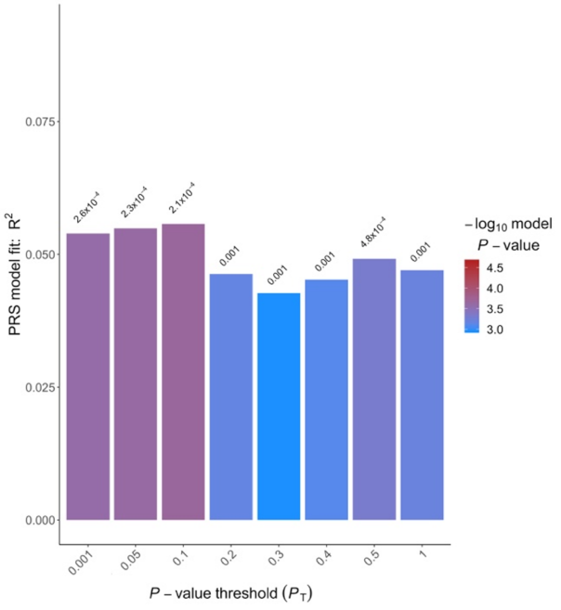

D) Sub-node D (most represented population: 49% White-Brazilian recruited in Brazil; 16% Brown-Brazilian recruited in Brazil; 7% Arab/Maghreb recruited in France)

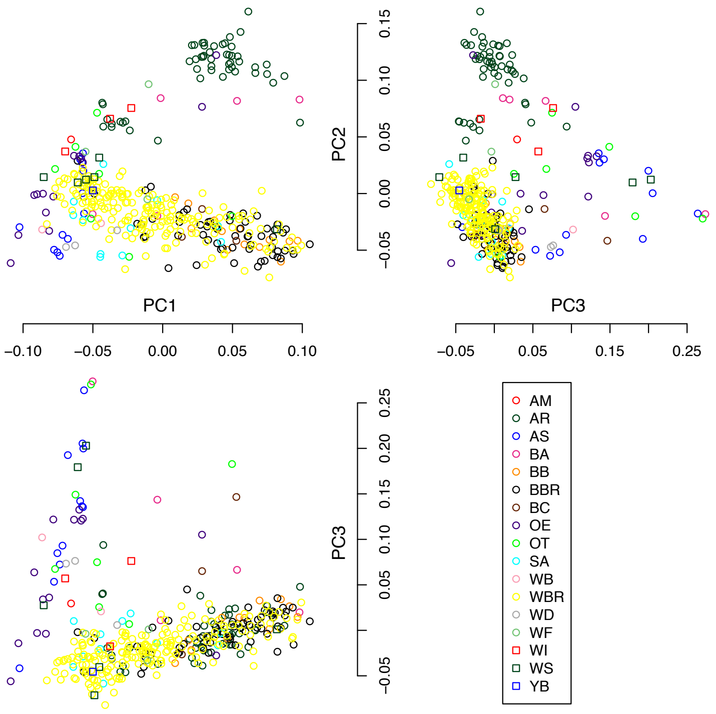

D) SZ-PRS - at Pt=0.05, R^2=0.131; p=3.2x10^{-8}

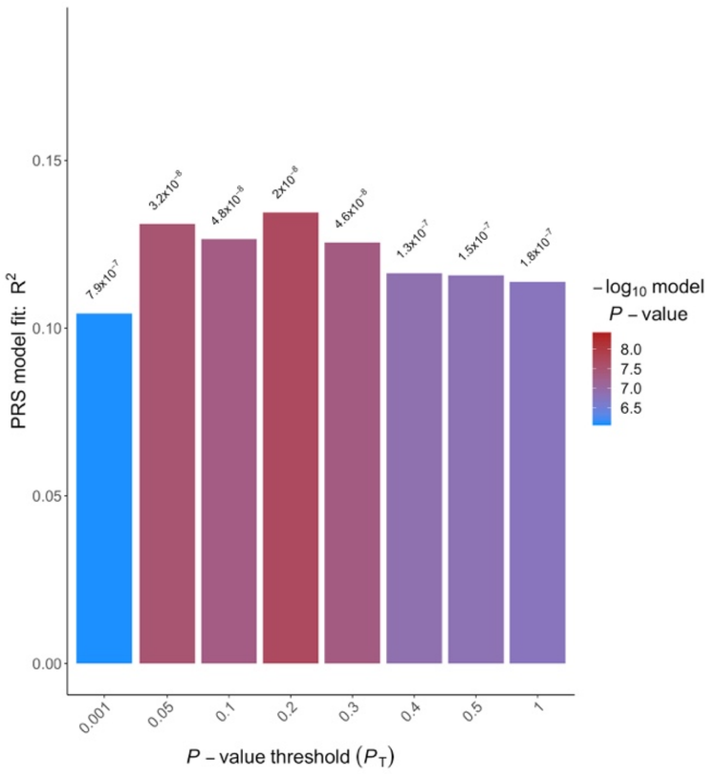

E) Sub-node E (most represented population Asian recruited in UK or Holland, Indian recruited in UK, American and South Americans recruited in Spain, Surinamese recruited in Holland, mixed White-Black Caribbean recruited in UK)

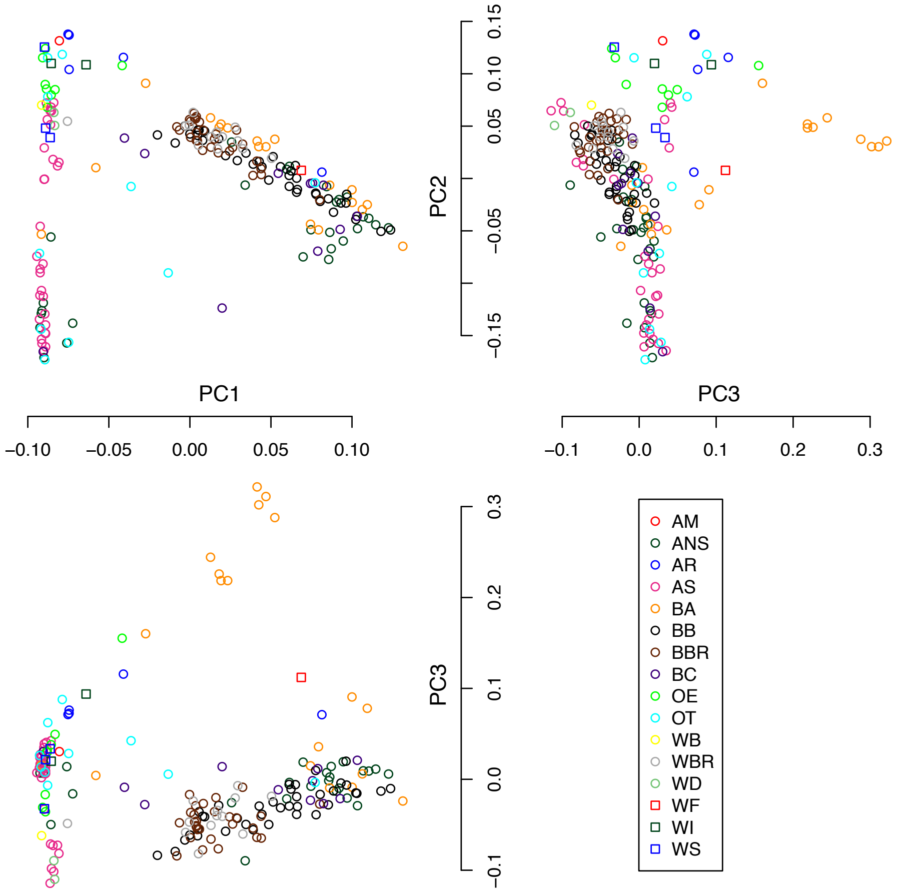

E) SZ-PRS - at Pt=0.05, R^2=0.06; p=0.004

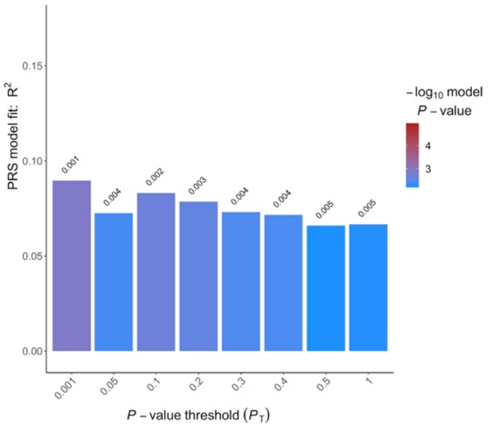

Sub-node F (most represented population Black African and Black Caribbean recruited in the UK)

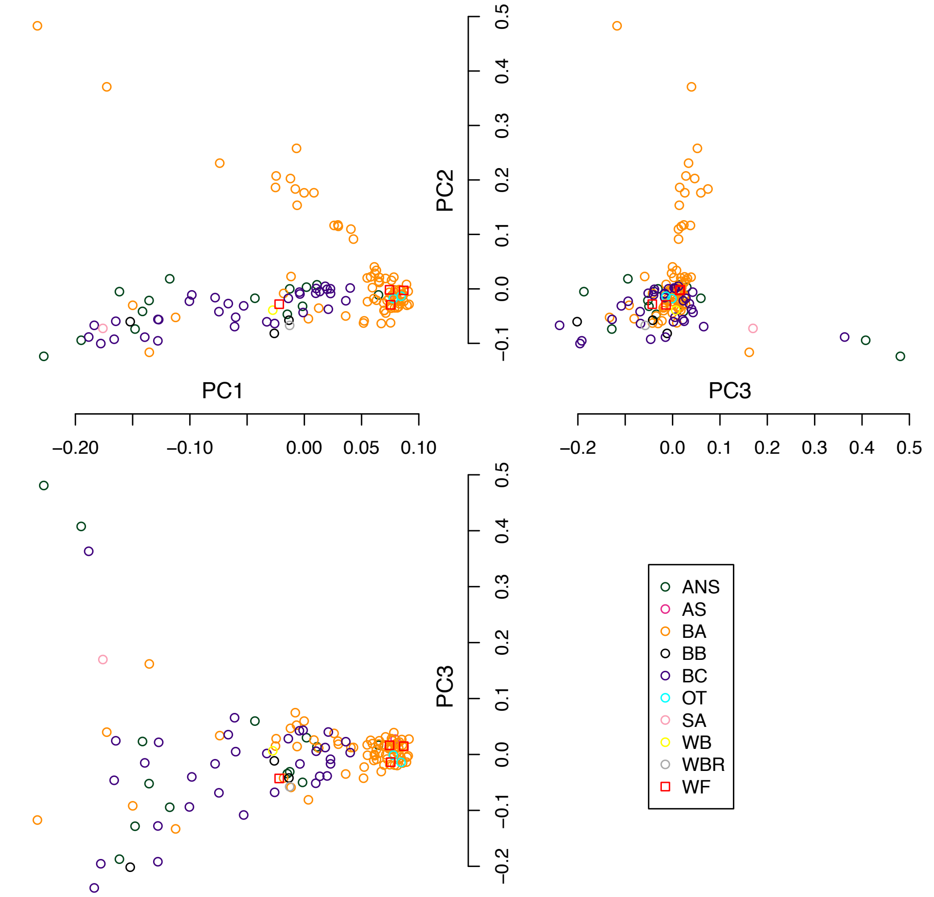

F) SZ-PRS - at Pt=0.05, R^2=NS

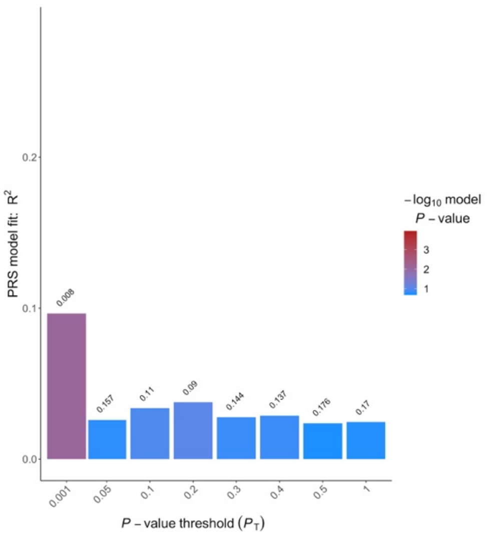

Figure S5. Cases and controls distribution across population clusters.

The plots show no observable systematic differences in the PCA ancestry distribution between cases and controls in population sub-nodes of interest (i.e., ‘sub-node A’, mostly composed of British and Dutch individuals; ‘sub-node B’, mostly composed of Spanish individuals; ‘sub-node C’, mostly composed of Italian individuals; ‘sub-node D’, mostly composed of Brasilian individuals) and the final merged sample.

Sub-node A

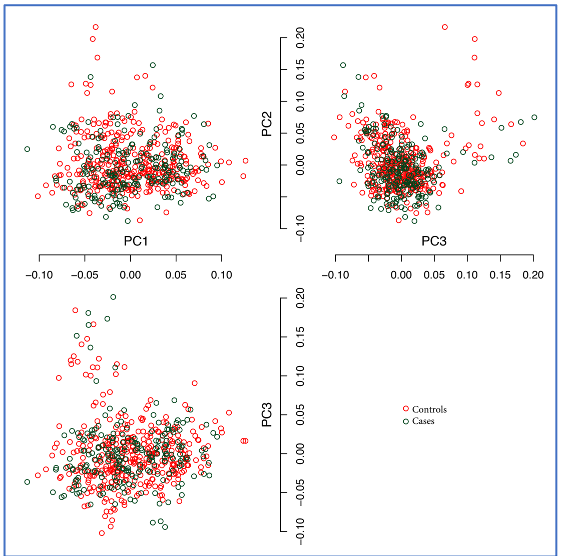

Sub-node B

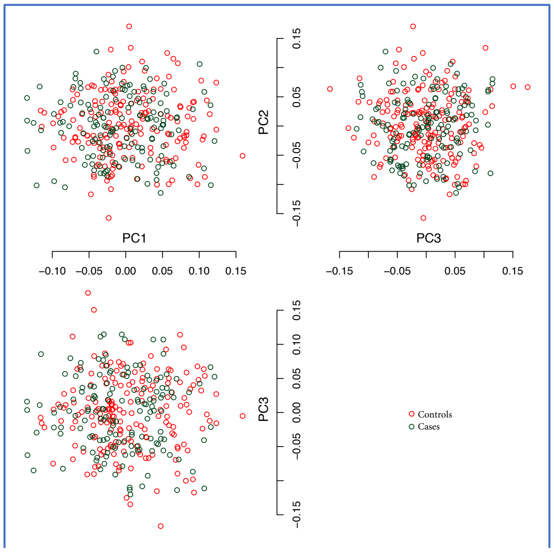

Sub-node C

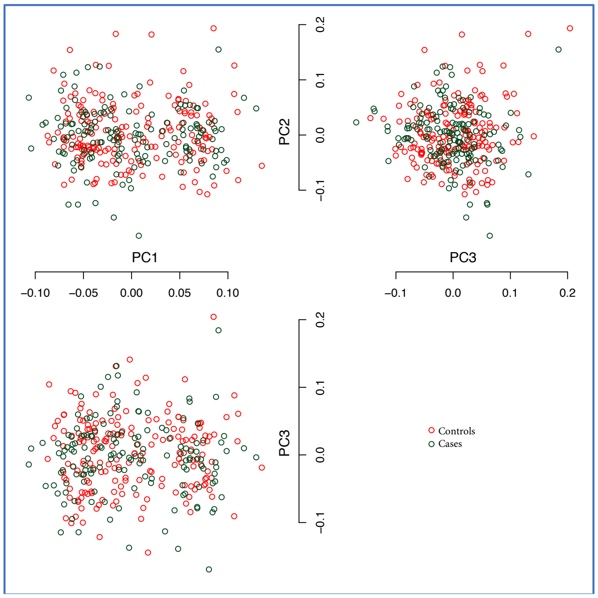

Sub-node D

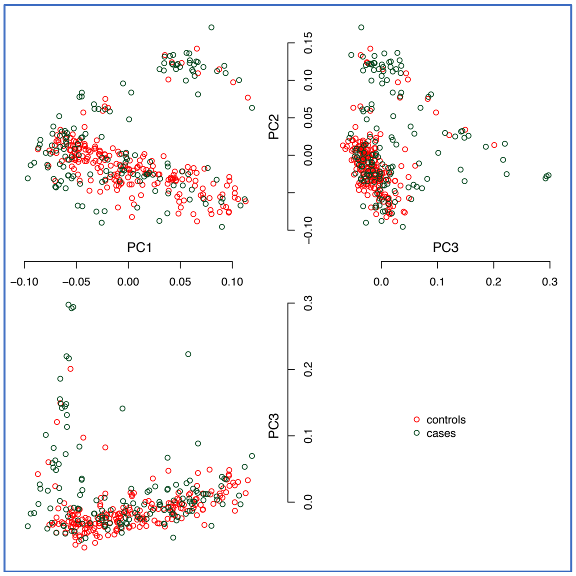

Final sample (subnodes A, B, C, and D)

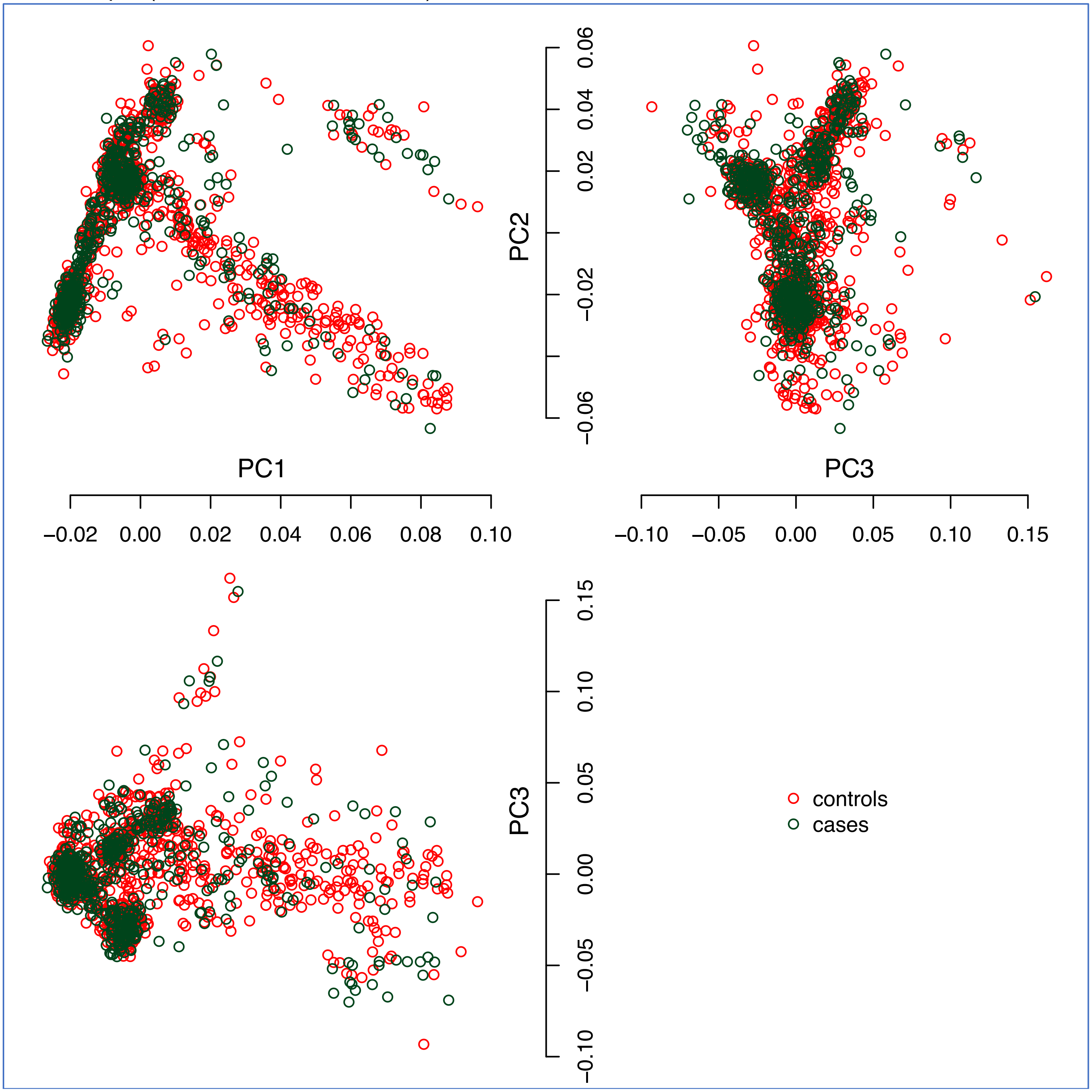

Figure S6. SZ-PRS in the final sample at different Pt-thresholds.

The plots show the variance in case-control status (y-axis) explained by SZ-PRS at different P_T (max no. of variants after clumping at P_T=1: 106,508; no. of variants at predefined P_T=0.05: 26,281). Permutation analysis confirmed that all the associations remained significant.

SZ-PRS

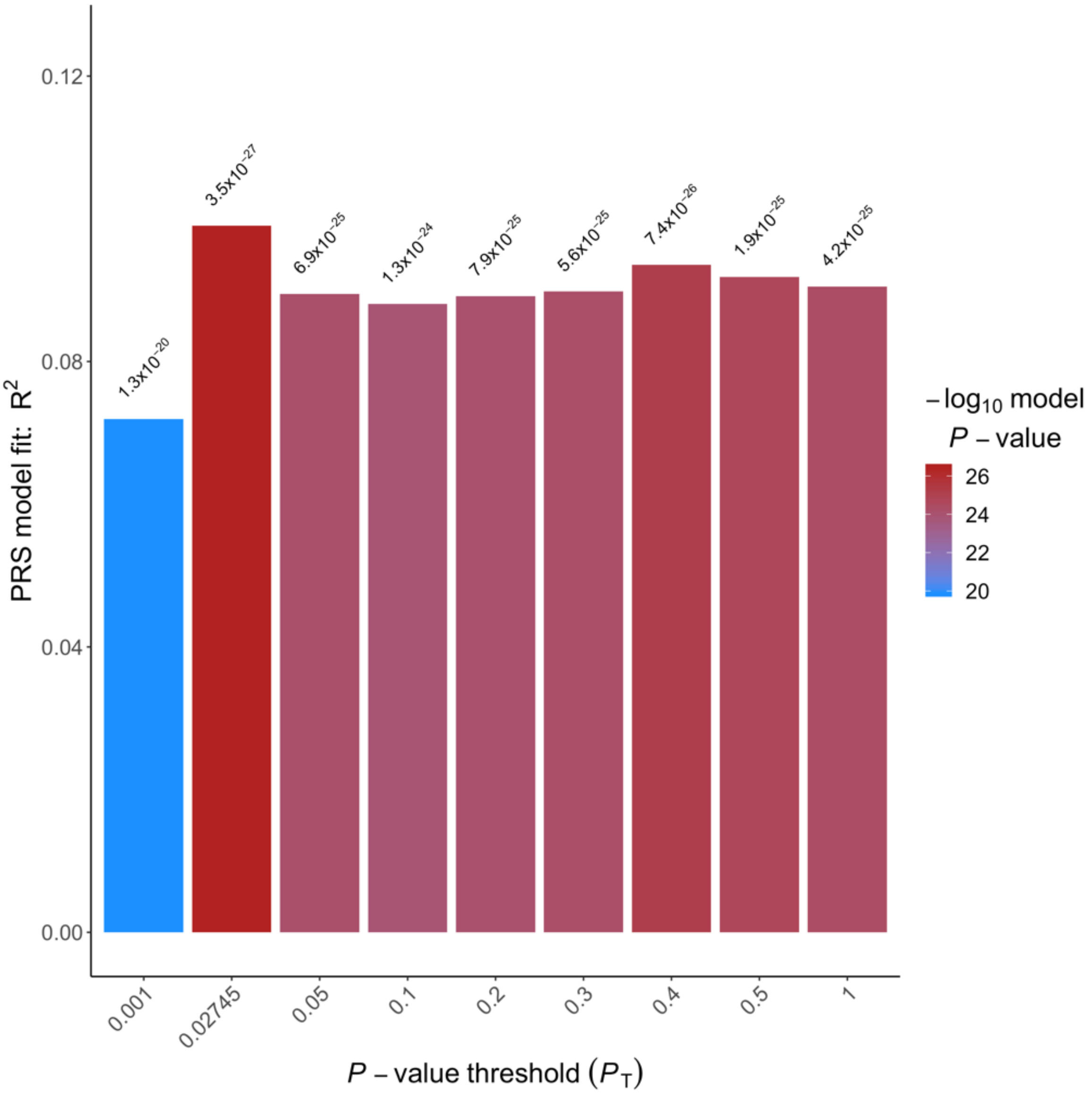

Figure S7. SZ-PRS by positive and negative symptom dimensions in patients and controls.

The plots show the variance in POS and NEG dimensions (y-axis), in cases and controls, explained by SZ-PRS at different P_T (x-axis). The max no. of risk variants after clumping at P_T=1 was 106,508; the no. of risk variants at predefined P_T=0.05 was 26,281. Permutation analysis in cases showed that associations remained significant after 5,000 permutations (empirical p-values at the best Pt threshold: POS=0.07; NEG=0.055).

Positive symptoms by SZ-PRS in cases

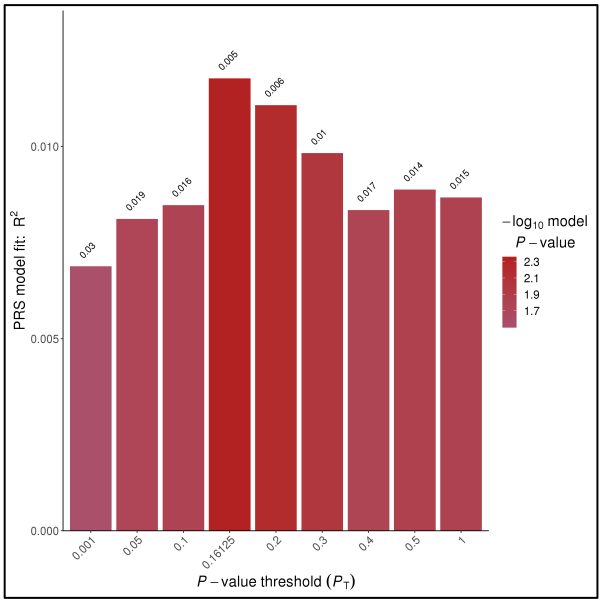

Negative symptoms by SZ-PRS in cases

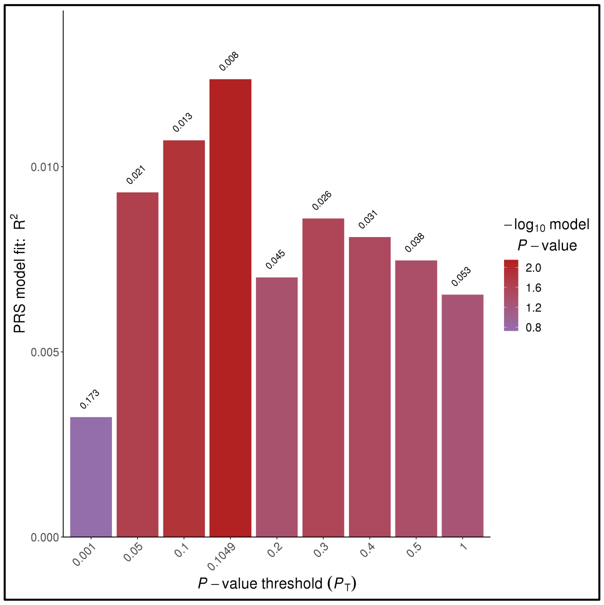

Positive psychotic experiences by SZ-PRS in controls

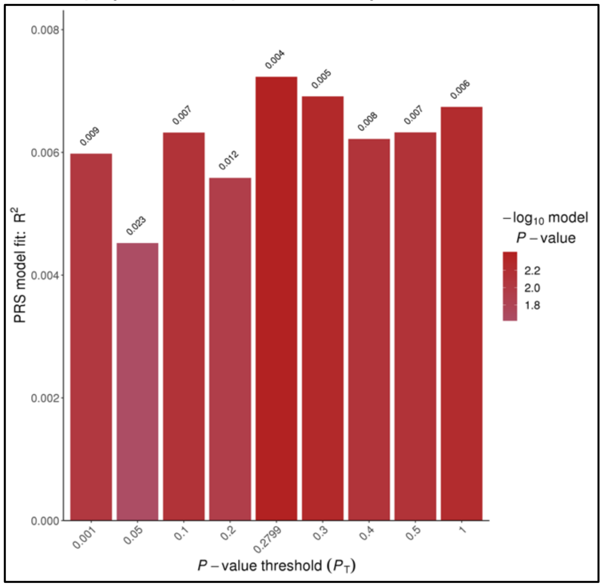

Negative psychotic experiences by SZ-PRS in controls

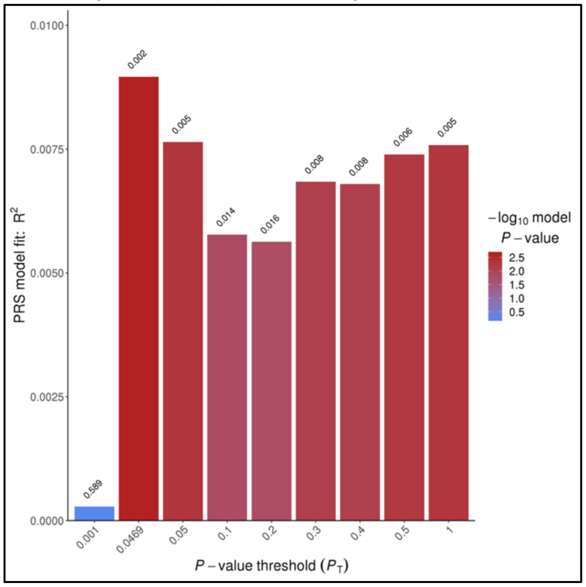

Table S2. Self-reported ethnicity of cases and controls in the final sample in each population cluster.

Subnode A

Self-reported Ethnicity	Freq.	Percent	Cum.
BA	5	0.88	0.88
JE	1	0.18	1.06
OE	48	8.50	9.56
OT	2	0.35	9.91
SA	1	0.18	10.09
WB	211	37.35	47.43
WD	225	39.82	87.26
WF	61	10.80	98.05
WIR	10	1.77	99.82
WS	1	0.18	100.00
Total	565	100.00	

Subnode B

Self-reported Ethnicity	Freq.	Percent	Cum.
AR	1	0.30	0.30
OE	9	2.73	3.03
SA	2	0.61	3.64
WB	1	0.30	3.94
WBR	12	3.64	7.58
WF	26	7.88	15.45
WPO	2	0.61	16.06
WS	277	83.94	100.00
Total	330	100.00	

Subnode C

Self-reported Ethnicity	Freq.	Percent	Cum.
AM	1	0.29	0.29
AR	1	0.29	0.58
BA	1	0.29	0.88
OE	25	7.31	8.19
WB	6	1.75	9.94
WBR	39	11.40	21.35
WD	1	0.29	21.64
WF	7	2.05	23.68

WI	261	76.32	100.00
Total	342	100.00	

Subnode D

Self-reported Ethnicity	Freq.	Percent	Cum.
AM	1	0.27	0.27
AR	50	13.33	13.60
AS	12	3.20	16.80
BA	5	1.33	18.13
BB	12	3.20	21.33
BBR	58	15.47	36.80
BC	2	0.53	37.33
OE	13	3.47	40.80
OT	7	1.87	42.67
SA	11	2.93	45.60
WB	2	0.53	46.13
WBR	187	49.87	96.00
WD	3	0.80	96.80
WF	3	0.80	97.60
WI	3	0.80	98.40
WS	5	1.33	99.73
YB	1	0.27	100.00
Total	375	100.00	

Abbreviations:

AM American
ANS Antillean/Surinamese
AR Arab/Maghreb
AS Asian
BA Black African
BB Black Brazilian
BBR Brown Brazilian
BC Black Caribbean
JE Jewish - Other European
OE Other European
OT Other Mixed
SA South American
WB White British
WBR White Brazilian
WD White Dutch
WF White French

WI	White Italian
WIR	White Irish
WPO	White Portugal
WS	White Spanish
YB	Yellow Brazilian

References supplementary material

1. Quattrone D, et al. (2019): Transdiagnostic dimensions of psychopathology at first episode psychosis: findings from the multinational EU-GEI study. *Psychol Med*. 49:1378-1391.
2. Quattrone D, et al. (2021): Daily use of high-potency cannabis is associated with more positive symptoms in first-episode psychosis patients: the EU-GEI case–control study. *Psychological Medicine*. 51:1329-1337.
3. Hancock GR, Mueller RO (2001): Rethinking construct reliability within latent variable systems. In: Cudek R, Du Toit S, Sorbom D, editors. *Structural Equation Modeling: Present and Future : a Festschrift in Honor of Karl Jöreskog*. Linconlnwood, IL: Scientific Software International, Inc., pp 195-216.
4. Genomes Project Consortium, et al. (2015): A global reference for human genetic variation. *Nature*. 526:68-74.
5. Chaichoompu K, et al. (2019): IPCAPS: an R package for iterative pruning to capture population structure. *Source Code Biol Med*. 14:2.
6. Prive F, Aschard H, Ziyatdinov A, Blum MGB (2018): Efficient analysis of large-scale genome-wide data with two R packages: bigstatsr and bigsnpr. *Bioinformatics*. 34:2781-2787.
7. Price AL, et al. (2008): Long-range LD can confound genome scans in admixed populations. *Am J Hum Genet*. 83:132-135; author reply 135-139.
8. Schizophrenia Working Group of the Psychiatric Genomics C (2014): Biological insights from 108 schizophrenia-associated genetic loci. *Nature*. 511:421-427.

Appendix, the Cannabis Experience Questionnaire used in the EU-GEI study

Cannabis Experiences Questionnaire

STUDIE: EU GEI	Date of Birth
Subject number: |__| EU |__|__| - |__|__|__|__|	|__|__|-|__|__|-| 1 | 9 |__|
Time interval:	Period – Replicat | 0 |__|-| 0 |__|
Interviewer:	Date |__|__|-|__|__|-| 2 | 0 |__|__|

Instructions to researcher: *Please tick boxes as appropriate to indicate patient's responses. Please be reminded that some questions allow for more than one response.*

15.1 Have you ever smoked/used cannabis? O1 Yes O0 No

Appendix, the Cannabis Experience Questionnaire used in the EU-GEI study

If answer is **NO,** *go to 15.17*

15.2 How old were you when you first tried cannabis? .... [|] years

15.3 Why did you first try cannabis? *(You can tick more than one box)***:**

a) My friends were using it. O1 Yes O0 No
b) My family members were using it O1 Yes O0 No
c) To feel better (to get relief from either physical or psychological discomfort) O1 Yes O0 No
d) Other *(please explain) (not for data entry)* O1 Yes O0 No

____

Instructions to researcher: *Please consider as current smokers all participants who report usually using/ smoking cannabis (incl. patients who have not smoked while inpatient/in prison and patients who report occasional use even if it is once every couple of years etc).*

15.4 Do you currently use cannabis? O1 Yes O0 No

If **Yes,** please answer *b*, if **No,** *go to 15.7*

b. If **YES**, why did you continue to use cannabis? *(You can tick more than one box)***:**

a) I like the effect, it gives me a buzz O1 Yes O0 No
b) It makes me feel relaxed O1 Yes O0 No
c) It makes me feel less nervous and anxious O1 Yes O0 No
d) It makes me feel more sociable O1 Yes O0 No
e) Other *(please explain)* O1 Yes O0 No

____

15.5 Would you like to stop using cannabis one day? O1 Yes O0 No

b. If yes, please explain *(not for data entry):*

___

15.6 Does/did cannabis affect your health in any way O1 Yes O0 No

b. If yes, please explain *(not for data entry)*:

___

15.7 If you are not a current user, how long ago did you stop smoking cannabis? [ ][ ] months

b. Why did you stop? please explain *(not for data entry)*:

___

15.8 How do/did you mostly use cannabis?

O1 I smoke/smoked it in a joint with tobacco
O2 I smoke/smoked it in a joint without tobacco
O3 I smoke/smoked it using a bong
O4 I eat/ate or drink/drank it
O5 Other (please explain): ___

15.9 How often do/did you use cannabis?

O1 Every day
O2 **(**More than) once a week
O3 A few times each month
O4 A few times each year
O5 Only once or twice

15.10 When do/did you mostly use cannabis?

O1 During the day
O2 During the evening
O3 During the day and evening
O4 At weekends
O5 During weekends and weekdays

15.11 Do you/did you mostly use cannabis:

O1 Socially (with friends)
O2 On my own

15.12 On average how much money per week do/did you usually spend on cannabis?

O1 Less than £2.50 (< €2.75)

O2 £2.50 - £5 (€2.75 - €5.50)

O3 £6 - £10 (€6.50 - €11)

O4 £11 - £15 (€12 – €16.50)

O5 £16 - £20 (€17.50 - €22)

O6 Above £20 (above €22)

15.13 What type of cannabis do/did you mostly use?

O1 Hash (cannabis resin/solid)

O2Imported herbal cannabis

O3Home-grown skunk/ Sensimilla

O4Super skunk

O5Other *(please state)*: ______________________________

15.14. Why did you choose the above type? ____

15.15. How often have you had these experiences while smoking cannabis?

Please rate whether it was a good, bad or neutral experience. If rarely or never, ignore rating (good, bad, neutral) and go to next item.

	Rarely or never	From time to time	Sometimes	More often than not	Almost always	Good	Bad	Neutral
a) Fearful	O0	O1	O2	O3	O4	O1	O2	O0
b) Feel like going crazy/mad	O0	O1	O2	O3	O4	O1	O2	O0
c) Nervy	O0	O1	O2	O3	O4	O1	O2	O0
d) Suspicious	O0	O1	O2	O3	O4	O1	O2	O0
e) Feeling happy	O0	O1	O2	O3	O4	O1	O2	O0
f) Full of plans/ideas	O0	O1	O2	O3	O4	O1	O2	O0
g) Hearing voices	O0	O1	O2	O3	O4	O1	O2	O0
h) Able to understand the world better	O0	O1	O2	O3	O4	O1	O2	O0
i) Seeing visions	O0	O1	O2	O3	O4	O1	O2	O0

15.16 Life Time Cannabis History questionnaire

Instructions to researcher: *Please hand this section over to participant for completion. Explain to participant how to complete this part by using (a) as an example: If you were smoking cannabis when you were 15, were smoking 2-3 joints per day on average, you usually smoked hash and you only smoked by yourself.*

a) AGE RANGE: 0-11

i. **Did you use cannabis between the ages of 0 and 11?** O1 Yes O0 No

ii. **Frequency**
- O1 Every day
- O2 More than once a week
- O3 About once a week
- O4 About once/twice a month
- O5 A few times a year
- O6 About once a year
- O7 I have only used cannabis once or twice

iii. **Quantity** *(average per day)*
- O1 1 Joint
- O2 2 or 3 Joints
- O3 4 or more Joints

iv. **Mostly shared** O1 Yes O0 No

v. **Type**
- O1 Hash (cannabis resin/solid)
- O2 Imported Herbal cannabis
- O3 Home-grown skunk/Sensimilla
- O4 Super skunk
- O5 Other (please state):_________________

vi. **Setting of use**
- O1 Socially (with friends)
- O2 On my own
- O3 Both

b) AGE RANGE: 12-16

i. **Did you use cannabis between the ages of 12 and 16?** O1 Yes O0 No

ii. **Frequency**
- O1 Every day
- O2 More than once a week
- O3 About once a week
- O4 About once/twice a month
- O5 A few times a year
- O6 About once a year
- O7 I have only used cannabis once or twice

**iii.**	**Quantity** *(average per day)*	O1 1 Joint<br>O2 2 or 3 Joints<br>O3 4 or more Joints
**iv.**	**Mostly shared**	O1 Yes O0 No
**v.**	**Type**	O1 Hash (cannabis resin/solid)<br>O2 Imported Herbal cannabis<br>O3 Home-grown skunk/Sensimilla<br>O4 Super skunk<br>O5 Other (please state):_________________
**vi.**	**Setting of use**	O1 Socially (with friends)<br>O2 On my own<br>O3 Both

c) AGE RANGE: ABOVE THE AGE OF 17

**i.**	**Did you use cannabis from the age of 17 onwards?**	O1 Yes O0 No
**ii.**	**Frequency**	O1 Every day<br>O2 More than once a week<br>O3 About once a week<br>O4 About once/twice a month<br>O5 A few times a year<br>O6 About once a year<br>O7 I have only used cannabis once or twice
**iii.**	**Quantity** *(average per day)*	O1 1 Joint<br>O2 2 or 3 Joints<br>O3 4 or more Joints
**iv.**	**Mostly shared**	O1 Yes O0 No
**v.**	**Type**	O1 Hash (cannabis resin/solid)<br>O2 Imported Herbal cannabis<br>O3 Home-grown skunk/Sensimilla<br>O4 Super skunk<br>O5 Other (please state):_________________
**vi.**	**Setting of use**	O1 Socially (with friends)<br>O2 On my own<br>O3 Both

d) If your pattern of cannabis use has changed overtime, please state why? (not for data entry)

____

e) Dependence screening for cannabis

Have you ever experienced 3 or more of the following characteristics?

	Lifetime		Last 12 months	
1. Tolerance, as defined by either of the following:				
a. A need for markedly increased amounts of the substance to achieve intoxication or desired affect.	O1 Yes	O0 No	O1 Yes	O0 No
b. Markedly diminished effect with continued use of the same amount of the substance	O1 Yes	O0 No	O1 Yes	O0 No
2. Withdrawal, as manifested by either of the following:				
a. The characteristic withdrawal syndrome for the substance	O1 Yes	O0 No	O1 Yes	O0 No
b. The same (or a closely related) substance is taken to relieve or avoid withdrawal symptoms	O1 Yes	O0 No	O1 Yes	O0 No
3. The substance is often taken in larger amounts or over a longer period than was intended	O1 Yes	O0 No	O1 Yes	O0 No
4. There is a persistent desire or unsuccessful efforts to cut down or control substance use	O1 Yes	O0 No	O1 Yes	O0 No
5. A great deal of time is spent in activities necessary to obtain the substance, use the substance, or recover from its effects	O1 Yes	O0 No	O1 Yes	O0 No
6. Important social, occupational, or recreational activities are given up or reduced because of substance use	O1 Yes	O0 No	O1 Yes	O0 No
7. The substance use is continued despite knowledge of having a persistent or recurrent physical or psychological problem that is likely to have been caused or exacerbated by the substance	O1 Yes	O0 No	O1 Yes	O0 No

15.17 Instructions to researcher: Please ask for each drug: ***Did you ever use?*** If **YES**, please continue the questions concerning current and past use. ***"Please also assess alcohol and drugs when applicable, (see separate Alcohol& Nicotine sheet)"***

[a] Dependence screening for any drugs:

1. Tolerance, as defined by either of the following:
    a. A need for markedly increased amounts of the substance to achieve intoxication or desired affect.
    b. Markedly diminished effect with continued use of the same amount of the substance
2. Withdrawal, as manifested by either of the following:
    a. The characteristic withdrawal syndrome for the substance
    b. The same (or a closely related) substance is taken to relieve or avoid withdrawal symptoms
3. The substance is often taken in larger amounts or over a longer period than was intended
4. There is a persistent desire or unsuccessful efforts to cut down or control substance use
5. A great deal of time is spent in activities necessary to obtain the substance, use the substance, or recover from its effects
6. Important social, occupational, or recreational activities are given up or reduced because of substance use
7. The substance use is continued despite knowledge of having a persistent or recurrent physical or psychological problem that is likely to have been caused or exacerbated by the substance

[b] Definition 'Abuse'

A. A maladaptive pattern of substance use leading to clinically significant impairment or distress, as manifested by one or more) of the following, occurring within a 12-month period:

1. Recurrent substance use resulting in a failure to fulfil major role obligations at work, school, or home
2. Recurrent substance use in situations in which it is physically hazardous
3. Recurrent substance-related legal problems
4. Continued substance use despite having persistent or recurrent social or interpersonal problems caused or exacerbated by the effects of the substance

B. The symptoms

The symptoms have never met the criteria for Substance Dependence for this class of substance
